# Tourists’ valuation of nature in protected areas: A systematic review

**DOI:** 10.1007/s13280-023-01845-0

**Published:** 2023-04-18

**Authors:** Milena Gross, Jasmine Pearson, Ugo Arbieu, Maraja Riechers, Simon Thomsen, Berta Martín-López

**Affiliations:** 1 Social-Ecological Systems Institute, Faculty of Sustainability Science, Leuphana University, Lüneburg, Universitätsallee 1, 21335 Lüneburg, Germany; 2 Senckenberg Biodiversity and Climate Research Centre, Frankfurt am Main, Georg-Voigt-Straße 14, 60325 Frankfurt am Main, Germany; 3 Smithsonian Conservation Biology Institute, National Zoological Park, 1500 Remount Road, Front Royal, VA 22630 USA; 4 Laboratoire d’Ecologie Systématique et Evolution, IDEEV, Université Paris-Saclay, Bât. 680 – 12, Route 128, 91190 Gif-sur-Yvette, France; 5 Institute of Ecology, Faculty of Sustainability Science, Leuphana University Lüneburg, Universitätsallee 1, 21335 Lüneburg, Germany

**Keywords:** Instrumental value, Intergovernmental Science-Policy Platform on Biodiversity and Ecosystem Services (IPBES), Intrinsic value, Relational value, The Economics of Ecosystems and Biodiversity (TEEB), Total Economic Value (TEV)

## Abstract

In the face of biodiversity loss, it is crucial to broaden the arguments for conservation of protected areas by acknowledging diverse values of nature. We systematically reviewed empirical studies to investigate tourists’ values of nature in protected areas over time and across regions. To do so, we explored (1) the main ecological and social characteristics of the case studies; (2) methodological approaches; and (3) value types. Based on the review of 152 articles, we found that economic valuation has received the most scientific attention, while socio-cultural valuation approaches have recently increased. Values were primarily elicited and analyzed quantitatively and in monetary metrics, although valuation methods and frameworks have diversified over the past two decades. However, considering the role of valuation methods and frameworks as value-articulating institutions, we suggest that future research on nature valuation also applies qualitative and non-monetary methods, elicits diverse values, and conducts plural valuation.

**Supplementary Information:**

The online version contains supplementary material available at 10.1007/s13280-023-01845-0.

## Introduction

Despite the fact that protected areas are at the core of conservation efforts to bend the curve of biodiversity loss (Díaz et al. 2020), there is increasing pressure to provide economic justification for their existence (West et al. 2006; Balmford et al. 2009; do Val Simardi Beraldo Souza et al. 2018). One of the pivotal economic arguments is that nature-based tourism in protected areas has globally become one of the most rapidly growing economic sectors (Davenport et al. 2002; Buckley 2009; Balmford et al. 2015; Buckley et al. 2019). For example, the number of touristic visits to terrestrial protected areas is estimated to be 8000 million per year, which generates approximately USD 600 000 million annually in direct in-country expenditures (Balmford et al. 2015). Moreover, Buckley et al. (2019) calculated the economic value of protected areas through the improved mental health of tourists, resulting in USD 6 000 000 million per year as a conservative global estimation. Research has often determined the economic value of nature-based tourism by estimating the travel costs, tourists’ willingness to pay to protect species, or to watch wildlife. For example, studies determined the economic value of wildlife species in Lake Nakuru National Park (Kenya) through the activity of tourism (Navrud and Mungatana 1994) and through tourists’ willingness to pay for the protection of the Ethiopian wolf (Estifanos et al. 2021). In addition, several studies suggest that raising public awareness about the economic benefits of tourism in protected areas can contribute to increasing the investment for their conservation (Balmford et al. 2002; Bruner et al. 2004; McCarthy et al. 2012; Waldron et al. 2013).

The positive impacts of tourism in protected areas include, but are not limited to economic benefits. Tourists assign value to nature in protected areas in more ways than only in economic terms, for example, by expressing recreational or aesthetic values (Harmon 2004). In fact, nature-based tourism in protected areas can contribute to nurturing tourists’ values of nature through direct experiences, such as recreational and touristic activities (Müller et al. 2019; Viirret et al. 2019) and aesthetic appreciation (Martínez Pastur et al. 2016; Gosal et al. 2018). For example, Yee et al. (2021) estimated the values of, among others, recreation and aesthetics in Tram Chim National Park (Vietnam) expressed by tourists. Conti and Lexhagen (2020) unraveled the recreational value of nature-based tourism in three Swedish national parks. In addition, researchers have demonstrated that these recreational and aesthetic experiences can promote people’s care and stewardship for nature and hence, its conservation (Soga and Gaston 2016; Schild 2019), and can also nurture social relationships, when they are enjoyed as a collective (Chan et al. 2016).

Therefore, nature-based tourism in protected areas does not only offer economic arguments for their conservation, but is also important for non-economic reasons. Indeed, if a tourist is asked to value a given experience with nature, they might assign different values to that experience depending on which valuation method is used and how. For example, by investigating the assessment of 17 empirical valuation studies, Jacobs et al. (2018) demonstrated that no single valuation method could cover the whole range of values of nature and that each valuation method was suitable to elicit particular value types. In addition, most value types are embedded in three prevalent valuation frameworks: i.e., Total Economic Value (TEV) (Krutilla 1967; Pearce and Turner 1990), The Economics of Ecosystem Services and Biodiversity (TEEB) (TEEB 2010), and the Intergovernmental Science-Policy Platform on Biodiversity and Ecosystem Services (IPBES) valuation framework (Díaz et al. 2015; Pascual et al. 2017). Therefore, the application of one or the other valuation framework also determines which values are elicited. For example, Martín-López et al. (2014) found that monetary valuation techniques embedded in TEV seemed to elicit the value of provisioning services, such as food and nature-based tourism, while socio-cultural valuation techniques promoted by TEEB appeared to unravel the value of regulating and cultural ecosystem services in Doñana Protected Area (Spain).

The TEV framework estimates the monetary value of nature by distinguishing between use and non-use values (Krutilla 1967; Pearce and Turner 1990). Use values refer to the consumption and enjoyment of nature through extractive and non-extractive experiences (i.e., direct use value); enjoyment of ecological processes (i.e., indirect use value); and potential use of nature in the future (i.e., option values). Non-use values refer to satisfaction that individuals derive from the existence of nature (i.e., existence value) and from knowing that other people can benefit from nature (i.e., bequest value) (Turner et al. 2003). The TEEB framework considers three value domains: biophysical, socio-cultural, and monetary (TEEB 2010). Finally, the IPBES framework conceptualizes three value types: nature as a means to human well-being (i.e., instrumental value); nature as an end in itself (i.e., intrinsic value); and the meaningfulness of human-nature relationships and social relationships mediated through nature (i.e., relational value) (Díaz et al. 2015; Pascual et al. 2017). Although these valuation frameworks are distinct, they can overlap since one value type embedded in a particular valuation framework might have a corresponding value in another. For example, Jacobs et al. (2018) found that the instrumental value (IPBES) was closely related with the direct and indirect use values (TEV) and economic value (TEEB).

Since tourists can value nature in protected areas in different ways, it is essential to understand how the choice of the valuation method(s) and framework influences the elicited values, which in turn can lead to different arguments for the conservation of protected areas. Therefore, the overall objective of our review is to assess how scientific studies have approached nature valuation by tourists in protected areas over time and across regions. Specifically, we seek to answer three research questions: (1) What are the main ecological and social characteristics of case studies on tourists’ values of nature in protected areas? (2) Which methods were applied to elicit and analyze tourists’ values of nature in protected areas? (3) Which values (embedded in the three valuation frameworks) of nature in protected areas were investigated for tourists in the scientific literature? Finally, we identify the key research gaps and priorities to foster future research on tourists’ valuation of nature in protected areas, which can ultimately provide insights for the conservation of protected areas.

## Methods

### Review process

**Fig. 1:**
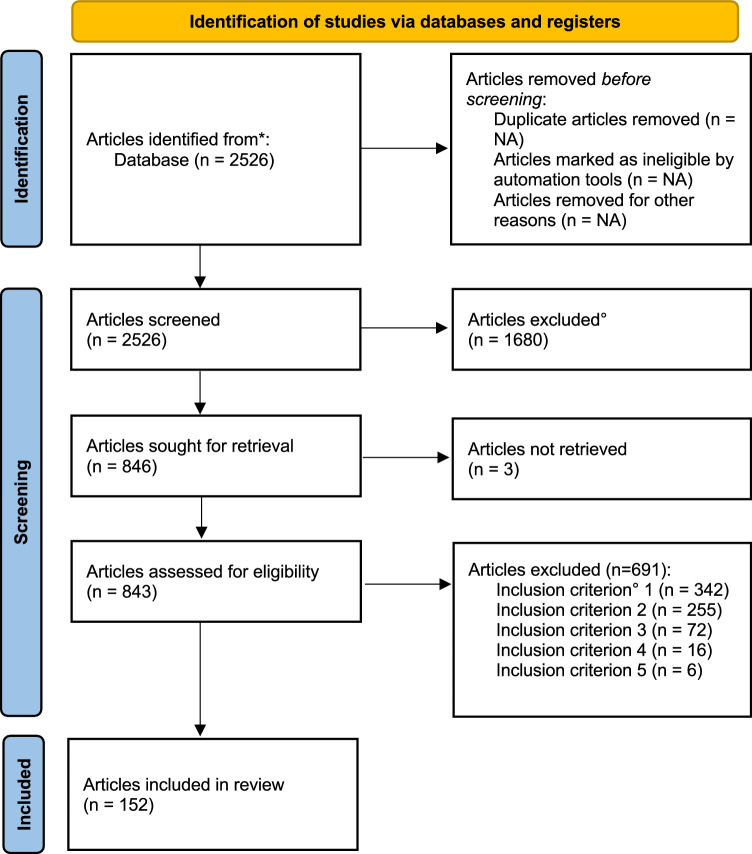
Flow diagram for the systematic review, based on Moher et al. (2009). * Search string (full search string in Supp. Material Table S2); *°* Inclusion criteria (logic behind the inclusion criteria in Supp. Material S3)

We conducted a systematic literature review^1^ of peer-reviewed scientific articles published in English on tourists' valuation of nature in protected areas, using the Scopus database. The review procedure followed the key steps recommended by Preferred Reporting Items for Systematic Reviews and Meta-Analyses (PRISMA) (Moher et al. 2009; Page et al. 2021) and the empirically validated review protocol by Luederitz et al. (2016) (Fig. 1, Supp. Materials S1 and S2).

The review followed a strict protocol of searching and inclusion criteria of articles to guarantee transparency and minimize bias. We chose the Scopus database in line with other systematic reviews in the field of human-nature interactions (Evers et al. 2018; Ferreira et al. 2020; Longato et al. 2021). We developed a search string that combined different terms related to tourists (i.e., touris* OR recreation* OR leisure OR travel* OR trip* OR journey*), value (i.e., *valu*), and protected areas (i.e., "protected area*" OR "protected landscape*" OR "biosphere reserve*" OR "nature reserve*" OR "natural monument*" OR "national park*" OR "natural park*" OR "biosphere area*" OR "conservation area*" OR "biosphere region*") (full search string in Supp. Material S2).

The search was applied to title, abstract, and keywords of peer-reviewed articles published in English until June 2021, resulting in 2526 articles. Although including scientific articles published in English can create a bias towards studies from countries with more resources for publishing in English-language journals, this is common for systematic reviews in the research field of human-nature interactions (Haase et al. 2014; Martín-López et al. 2019; Johnson et al. 2022; Vercillo et al. 2022). To ensure scientific quality based on peer-review process, we did not consider book chapters and conference proceedings (Haase et al. 2014; Martín-López et al. 2019; Johnson et al. 2022).

To identify relevant studies out of the 2526 articles, we applied five inclusion criteria. We selected articles that empirically (criterion 1) investigated values (criterion 2) of nature (criterion 3) in protected areas (criterion 4) expressed by tourists (criterion 5). To guarantee the use of empirical data (criterion 1) and to avoid redundancy (Pullin and Stewart 2006), we excluded reviews (five inclusion criteria described in Supp. Material S3).

We identified 846 articles as eligible for full text screening after examining the title and abstract based on the above-mentioned five criteria. If the full text was not accessible, we contacted authors of the respective articles through email and ResearchGate,^2^ if possible. Due to lack of access, we excluded three articles. After screening the full text of the remaining 843 articles, 152 articles fulfilled the five inclusion criteria and hence, were selected for in-depth analysis. Supp. Material S4 presents the list of articles considered in the systematic review.

Following a deductive-inductive two-step procedure, we developed a coding scheme to organize the database. We identified five sets of variables that represent the main topics of this review: (1) meta-information of the article; (2) biogeographical information of the empirical case studies; (3) valuation information; (4) information on data gathering and analysis; and (5) information on values. The final coding scheme consisted of 18 categories of variables. Information on categories (e.g., type of ecosystem) that could not be retrieved from the article was reinvestigated through searching reliable online sources. To categorize the value type and valuation framework, we extracted the information directly from the article. However, for those articles that did not explicitly state the value type(s) or valuation framework(s) used, we assigned them a posteriori. For example, if an article applied contingent valuation to estimate the willingness to pay to visit a protected area, we considered the value as economic value (TEEB). This economic value could also be classified as instrumental value (IPBES). However, since willingness to pay does not necessarily refer to the importance of nature as a means to an end (i.e., instrumental value), we classified willingness to pay as economic value only. Regarding non-economic values, for which the valuation framework was not stated, we generally classified them as a socio-cultural value (TEEB) (Rossi et al. 2020). Only if more information was given in the paper, we assigned a specific value type. For instance, we classified tourists’ values regarding opportunities for recreation as recreational value (Müller et al. 2019). Since specific information on value types and frameworks was lacking for most articles, our results might therefore be biased towards TEEB (coding scheme described in Supp. Materials S5 and S6). In addition, we conducted qualitative content analysis to associate the value types with the three valuation frameworks. Supp. Material Table S10 presents quotes from the reviewed articles that reflect the association.

Five authors were involved in the coding. We accounted for inter-coder reliability by taking three measures. First, each author coded five articles individually. Then, results and questions were collaboratively discussed to deepen the mutual understanding of the coding scheme and to maximize coherence in the coding process. Second, the remaining articles were distributed among the five authors who then coded each article individually. Each author stated their coding confidence for each of the five sets of categories and added remarks and questions, if any. The first author reviewed them and discussed them with the other authors, if necessary. Third, the first author reviewed the data entries for coherence.

### Data synthesis and quantitative analysis

For the variables used to answer the three research questions, we calculated the frequencies of articles (Table 1). In addition, we used the variables ‘location of protected area’ and ‘latitude and longitude of the protected area’ to spatially represent the scientific literature on tourists’ valuation of nature in protected areas. Moreover, we compared the regional share of articles found in this review with the relative share of protected areas worldwide. To do so, we collected the information on protected areas worldwide from the World Database on Protected Areas (WDPA) (IUCN and UNEP-WCMC 2022).

**Table 1:** Overview of the variables analyzed to meet the overall objective and answer the research questions

Research questions	Variables analyzed
Meta-information of nature valuation by tourists in protected areas over time and across regions	Year of publication; Region of protected area
(1) Main ecological and social characteristics	Ecosystem of protected area; Object of value: nature; Object of value: non- natural elements Target social group; Main objective of article to conduct valuation
(2) Methods applied to elicit and analyze values	Classification of valuation technique/method; Broad method of valuation; Specific valuation method; Data type in value elicitation process; Value metric in value elicitation process; Data type in data analysis; Value metric in data analysis
(3) Values investigated	Value type

We conducted a correspondence analysis (CA) with FactorMineR package (Lê et al. 2008) in RStudio (RStudio Team 2021) to explore the relationship between valuation methods and value types. CA allows investigating relationships among qualitative variables and has been broadly used in the field of human-nature interactions (Teixeira et al. 2019; Valente et al. 2020; Cifuentes-Espinosa et al. 2021). We excluded data for which the frequency was lower than four cases (e.g., narrative analysis (*n* = 3) and instrumental value (*n* = 2)) to reduce biases. This exclusion process was adopted in other multivariate statistics to explore relationships between variables in systematic reviews (Quintas-Soriano et al. 2022). We visualized the CA results in a biplot representing the scores of the first and second axes.

## Results

### Temporal trend and regional distribution

**Fig. 2:**
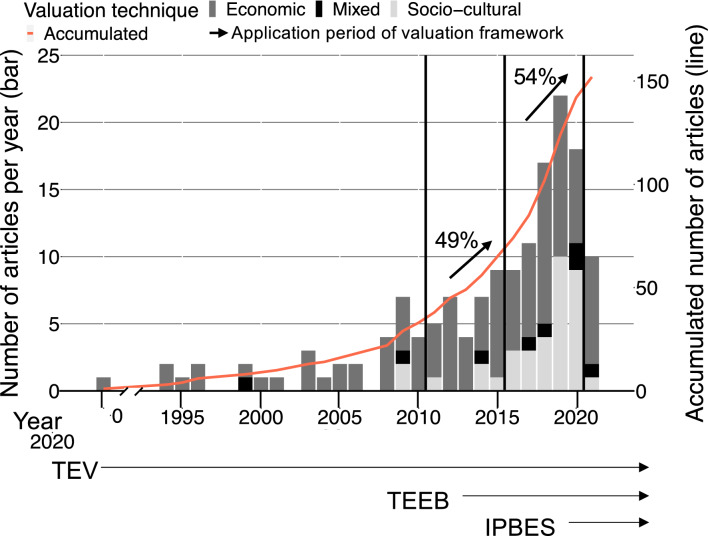
Temporal distribution of 152 articles published on tourists’ valuation of nature per year (bars and left *Y* axis) and accumulated number of articles (red line and right *Y* axis). Since we considered articles published until 23 June, 2021 (when the search was done), the actual number of articles issued in 2021 might be higher. *TEV* the Total Economic Value; *TEEB* The Economics of Ecosystems and Biodiversity; *IPBES* the Intergovernmental Science-Policy Platform on Biodiversity and Ecosystem Services

We identified an increasing trend of empirical research on tourists’ valuation of nature in protected areas from 1977 to 2021, in which different valuation techniques were used (Fig. 2). Economic valuation (*n* = 108 of all articles; 71%) was most frequently applied compared to socio-cultural valuation (*n* = 36; 24%) and a mixed approach (*n* = 8; 5%). Based on the release dates of the three valuation frameworks (i.e., TEV, TEEB, and IPBES), we distinguished three periods (Fig. 2). The first period (1977–2010), influenced by TEV, comprised 33 articles that mainly applied economic valuation (91%). The second period (2011 to 2015), influenced by TEEB, comprised 32 articles that mainly used economic valuation (84%). The third period (2016-2020), influenced by IPBES, included 77 articles. In this period, we found a remarkable increase of articles using socio-cultural valuation (38%). The number of articles in the second period increased by +49% compared to the previous five years (2006–2010) and by +54% in the third compared to the second period (Fig. 2).

**Fig. 3:**
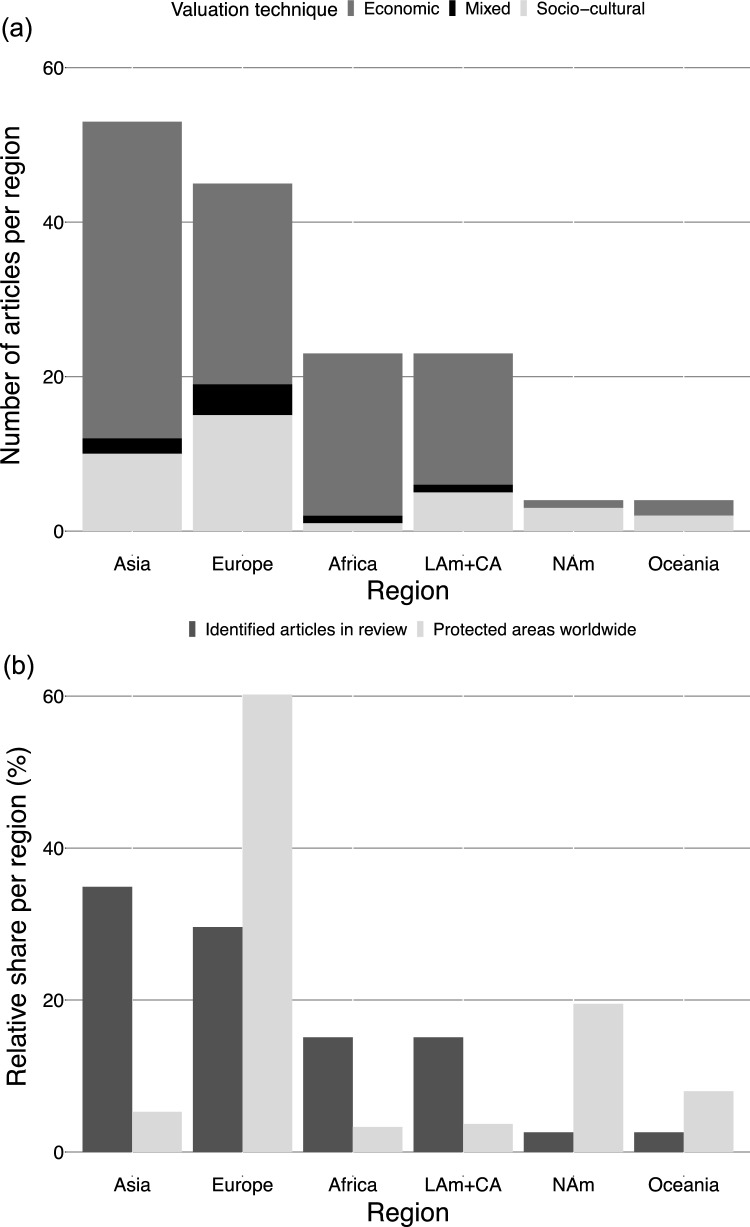
Regional distribution of 152 articles published on tourists’ valuation of nature. (**a**) Absolute number of articles per region and valuation techniques applied. (**b**) Regional share (%) of identified articles in this review (dark grey) and of protected areas worldwide (light grey). Information on numbers of protected areas worldwide was sourced from the World Database on Protected Areas (WDPA) (IUCN and UNEP-WCMC 2022). *LAm+CA* Latin America and the Caribbean; *NAm* North America

Tourists’ valuation of nature in protected areas was researched across the major regions of the world, but with an uneven distribution (Fig. 3a). Many articles focused on protected areas in Asia (*n* = 53 articles; 35% of all articles) and Europe (*n* = 45; 30%), while only four articles (3%) targeted protected areas in North America, although these articles investigated up to 38 protected areas (geographical overview of the protected areas represented in Supp. Material Fig. S1). Comparing the regional share of articles with the number of protected areas per region, tourists’ valuation of nature was more studied in Asia, Africa, and Latin America and the Caribbean, but less in Europe, North America, and Oceania (Fig. 3b). Economic and socio-cultural valuation techniques were applied across all regions but with some regional differences (Fig. 3a). While tourists’ value of nature in African protected areas was rarely studied with socio-cultural valuation methods (*n* = 1; 4% of all methods used in Africa), these techniques were applied above average in Europe (33%; *n* = 15), North America (75%; *n* = 3), and Oceania (50%; *n* = 2). Economic valuation was the most frequently applied technique in Asia (77%; *n* = 41 of all methods used in Asia), Europe (58%; *n* = 26), Africa (91%; *n* = 21), and Latin America and the Caribbean (74%; *n* = 17).

### Ecological and social characteristics of case studies

**Fig. 4:**
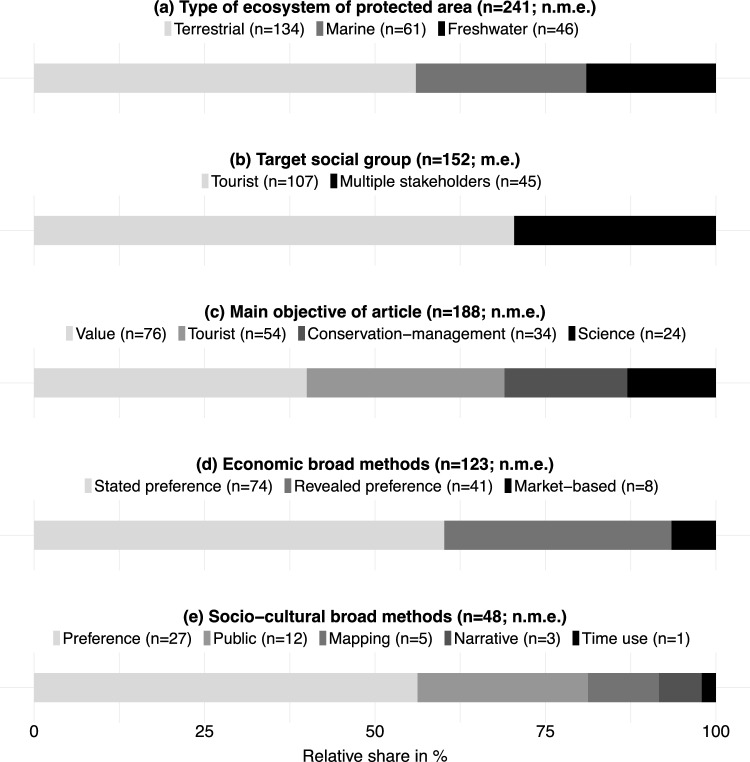
Descriptive overview of the ecological and social characteristics of the valuation exercises (**a**–**c**) and broad methods of articles reviewed (**d**-**e**), as resulted from the qualitative content analysis of *n* = 152 articles. (**a)** Type of ecosystem. (**b)** Target social group. (**c**) Main objective of the article. Labels of the main objective of articles indicate their orientation (e.g., ‘value’ means value-oriented). (**d)** Economic valuation methods applied. (**e)** Socio-cultural valuation methods applied.* Preference* Preference assessment;* Public* Public methods;* Mapping* (Participatory) Mapping;* Narrative* Narrative analysis. The broad socio-cultural method(s) of three articles could not be specified and thus, they are not displayed. Absolute frequency (n) and relative share (bar) of each (sub-)category are presented. *n* absolute frequency of (sub-)category; *n.m.e.* Data are not mutually exclusive; *m.e.* Data are mutually exclusive

For ecological characteristics, we found that tourists’ valuation took place in protected areas with various ecosystems. Terrestrial ecosystems were more studied (*n* = 134; 88%) than marine (*n* = 61; 40%) and freshwater ecosystems (*n* = 46; 30%) (Fig. 4a).

We also found that the natural element mostly chosen as object of valuation was the protected area itself (*n* = 100; 21% of all investigated natural elements), followed by cultural ecosystem services/non-material nature’s contributions to people (NCP) (*n* = 61; 13%), land-/seascape (*n* = 29; 6%), and iconic animal species (*n* = 20; 4%) (Supp. Material Table S7). Additionally, 41 articles (27%) valued elements that are not derived from nature, such as interpretive signs and improved walking trails (Supp. Material Table S8).

Regarding the social characteristics of the studies, 70% (*n* = 107) of the articles only targeted tourists, whereas the remaining articles (*n* = 45; 30%) elicited the value(s) of tourists and other stakeholders, such as local people and conservation managers (Fig. 4b). Moreover, we identified that the main objective of the studies was primarily value-oriented (n=76; 40% of all investigated main objectives) and tourist-oriented (*n* = 54; 29%), followed by conservation-management-oriented (*n* = 34; 18%) and science-oriented (*n* = 24; 13%) (Fig. 4c).

### Methodological approaches of the case studies

#### Valuation methods

Economic valuation studies applied three different families of methods (i.e., broad methods): stated preference (*n* = 74; 60% of all economic broad methods applied), revealed preference (*n* = 41; 33%), and market-based methods (*n* = 8; 7%) (Fig. 4d). Within stated preference methods, 57 articles (46% of all economic specific methods applied) used contingent valuation and 17 articles applied choice experiment (14%) (Supp. Material Table S9)

Concerning socio-cultural valuation, articles used five broad methods: preference assessment (*n* = 27; 53% of all socio-cultural broad methods applied), public methods (*n* = 12; 23%), (participatory) mapping (*n* = 5; 10%), narrative analysis (*n* = 3; 6%), and time use method (*n* = 1; 2%) (Fig. 4e). The specific method used within preference assessment was ranking/rating (*n* = 27; 50% of all socio-cultural specific methods applied). Within the broad method of (participatory) mapping, three out of five articles specifically applied Public Participation Geographic Information Systems (PPGIS). Regarding public methods, studies applied photo-series analysis (*n* = 12; 22%) and netnography (*n* = 3; 6%) alongside photo-series analysis.

Regarding mixed approaches, only eight articles (5% of all articles) applied both economic and socio-cultural valuation. There were few cases in which two families of broad economic or socio-cultural methods were applied. Nine articles (8% of all articles that applied economic valuation) applied two broad economic methods in the same study: stated preference and revealed preference methods (*n* = 6), stated preference and market-based methods (*n* = 2), and revealed preference and market-based methods (*n* = 1). Five articles (14% of all articles that applied socio-cultural valuation) applied two socio-cultural broad methods: (participatory) mapping and preference assessment (*n* = 3), narrative analysis and public methods (*n* = 1), and narrative analysis and preference assessment (*n* = 1).

#### Data collection and analysis

**Fig. 5:**
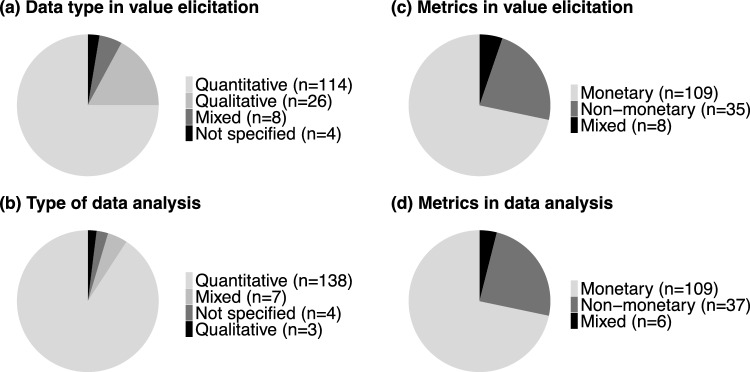
Descriptive overview of (**a)** data type and (**b)** metrics in value elicitation, (**c)** type of data analysis and (**d)** metrics in data analysis, as resulted from the qualitative content analysis of *n* = 152 articles. Data are mutually exclusive. *n* absolute frequency of sub-category

Most articles collected quantitative data (*n* = 114; 75% of all articles), followed by qualitative data (*n* = 26; 17%) and both data types (*n* = 8; 5%) (Fig. 5a). Similarly, the majority of articles also conducted quantitative data analyses (*n* = 138; 91%), followed by mixed (i.e., quantitative and qualitative; *n* = 7; 5%) and qualitative data analyses (*n* = 3; 2%) (Fig. 5b). Some articles that collected qualitative data transformed the data to run quantitative analyses. For example, qualitatively extracted information from Likert scales was translated into quantitative information to compute statistical analyses, such as ANOVA test (e.g., van Marwijk et al. 2012) and Spearman rho correlation (e.g., Chakrabarty et al. 2019).

Most articles elicited values in monetary metrics (*n* = 109; 72%), followed by non-monetary (*n* = 35; 23%) and both metrics (*n* = 8; 5%) (Fig. 5c). The metric was usually not changed between the data collection and data analysis with a few exceptions (Fig. 5c, d). For example, Chen et al. (2019) and Zhang et al. (2019) asked respondents to rank social values by distributing monetary values, which were then converted to non-monetary values in the data analysis.

### Value types and valuation frameworks

**Fig. 6:**
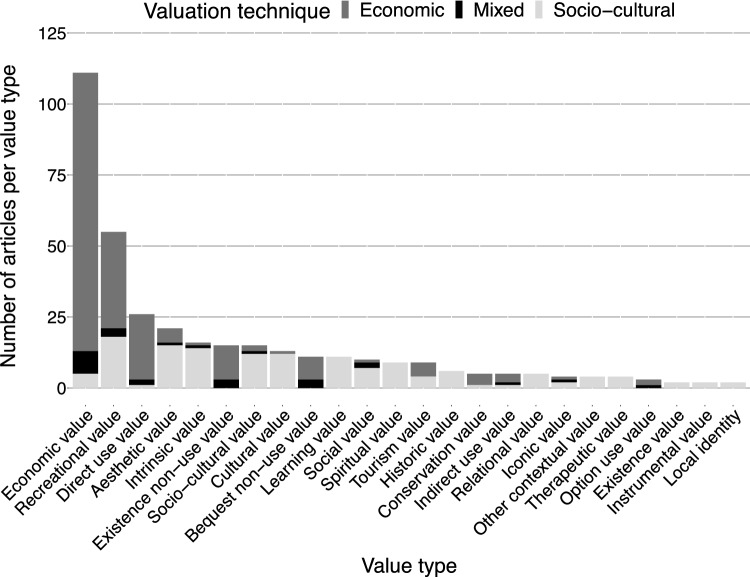
Descriptive overview of value type and valuation techniques, as resulted from the qualitative content analysis of *n* = 152 articles. The different valuation techniques applied per value type are indicated with the different colors in the bars. ‘Other contextual values’ refers to, for instance, future (*n* = 2) and eudemonic value (*n* = 1). Data are not mutually exclusive

**Fig. 7:**
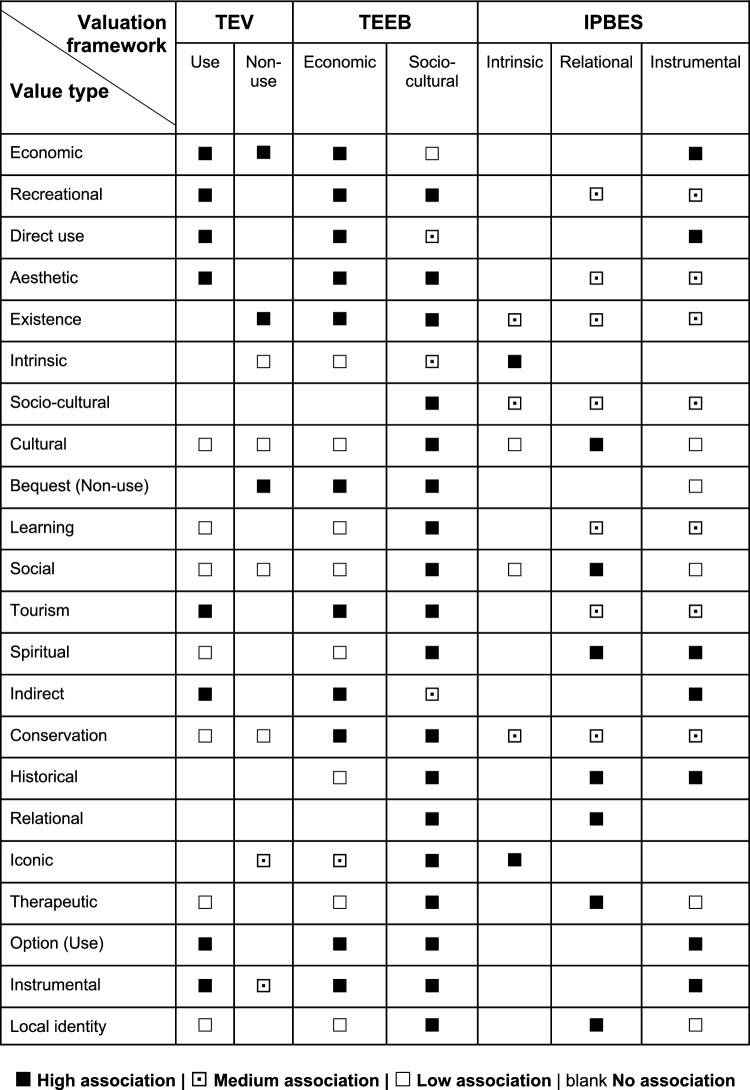
Association between value types identified in the literature review on tourists’ valuation of nature in protected areas and the value types embedded in the valuation frameworks of the Total Economic Value (TEV), The Economics of Ecosystems and Biodiversity (TEEB), and the Intergovernmental Science-Policy Platform on Biodiversity and Ecosystem Services (IPBES). Biophysical values according to TEEB are not considered because they cannot be expressed by tourists. Supp. Material Table S10 presents the quotes found in the literature of this systematic review that prove the association between value types and valuation frameworks as found in the reviewed articles

Literature on tourists’ valuation of nature in protected areas referred to 24 different value types (Fig. 6). Economic value (*n* = 111; 30% of all identified value types) received the most scientific attention, followed by recreational (*n* = 55; 15%), direct use (*n* = 26; 7%), and aesthetic values (*n* = 21; 6%). Importantly, when eliciting specific value types, articles did not explicitly refer to a valuation framework. Yet, the different value types identified in the review could be associated with different value types embedded in the three valuation frameworks (i.e., TEV, TEEB, and IPBES) (Fig. 7). For example, recreational value could be associated with value types within all three valuation frameworks, that is, direct use value (TEV), economic and socio-cultural values (TEEB), and relational and instrumental values (IPBES). Supp. Material Table S10 shows a compilation of exemplary quotes found in the reviewed literature that represent the associations between value types and valuation frameworks.

Most value types were elicited through both economic and socio-cultural valuation techniques. However, there were specific value types that were elicited by applying particular socio-cultural valuation techniques (Fig. 6). For example, spiritual, historic, and therapeutic values were elicited by applying only ranking (e.g., Apps et al. 2019; Chen et al. 2019) and mapping (e.g., Zhang et al. 2019). Although economic value was mainly elicited with economic valuation techniques, there were studies that applied socio-cultural valuation to uncover tourists’ economic value of nature in protected areas using the specific methods of ranking (e.g., Apps et al. 2019; Chen et al. 2019), rating (e.g., Mrotek et al. 2019), and mapping (e.g., Zhang et al. 2019). Moreover, value types that were originally embedded within TEV framework were primarily elicited with economic valuation techniques, with one notable exception: Apps et al. (2019) used the TEV framework, while applying the socio-cultural method of preference assessment. The application of socio-cultural valuation methods to elicit TEV values illustrates the connection between TEV and the socio-cultural value embedded in the TEEB framework (Fig. 7). For example, the direct use value of recreation (TEV) can be elicited by asking how much additional time a tourist is willing to travel to reach a protected area (e.g., Heyes and Heyes 1999), which represents a non-monetary metric and is considered a socio-cultural value under TEEB. See Supp. Material Table S10 for the specific quote that demonstrates this association.

### Relations between value types and valuation methods

**Fig. 8:**
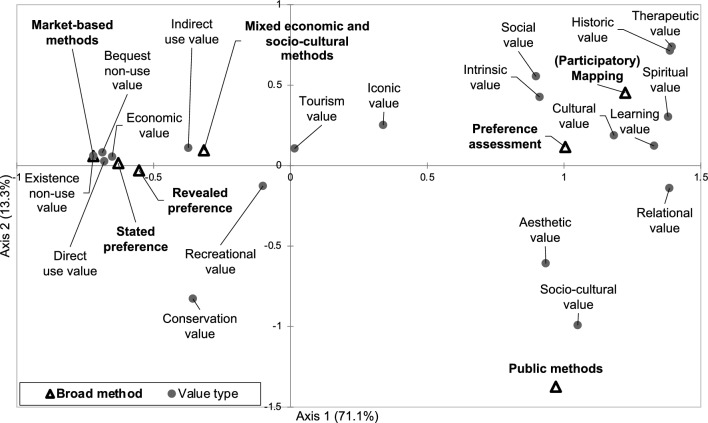
Biplot of the first two axes of the correspondence analysis showing the relations between broad families of valuation methods and value types. Data for which the number of sub-categories was lower than four cases (narrative analysis (*n* = 3) and instrumental value (*n* = 2)) were excluded to reduce biases

The correspondence analysis showed relations between broad families of valuation methods and specific value types, indicating a clear separation between economic and socio-cultural valuation methods and the value types elicited (Fig. 8). The first two factorial axes of the CA accumulated 84.4% of the total inertia. The first axis (71.1% of inertia) separated the two families of methods (i.e., economic and socio-cultural valuation methods) and specific value types. The negative scores of axis 1 represented economic valuation techniques and TEV, economic, and conservation values. The positive scores of axis 1 portrayed socio-cultural valuation techniques and many non-economic values, such as therapeutic, historical, relational, learning, and cultural value (Fig. 8). The second axis (13.3% of inertia) distinguished between the application of (participatory) mapping and public methods, both socio-cultural methods. The positive scores represented the application of (participatory) mapping and the elicitation of therapeutic, historical, and social values, whereas the negative scores portrayed the application of public methods and the elicitation of socio-cultural, conservation, and aesthetic values.

## Discussion

### Trends, distribution, and research gaps in tourists’ valuation of nature in protected areas

#### Tourists’ valuation of nature in protected areas over time

Our results demonstrate an increase of studies on tourists’ valuation of nature in protected areas over the last decade (Fig. 2). This increasing trend is consistent with previous systematic reviews focusing on valuation of ecosystem services/nature’s contributions to people (Haase et al. 2014; Acharya et al. 2019; Rau et al. 2020). Moreover, while previous research reviewing valuation of ecosystem services/nature’s contributions to people showed a stabilization of publications since 2015 (Martín-López et al. 2019), our review indicates an increasing trend in recent years of +54% in the last five years.

The temporal trend identified in this review also shows that economic valuation was predominant until 2015 and that there was an increase of socio-cultural valuation over the last five years (Fig. 2). These findings align with previous reviews demonstrating a dominance of economic valuation until 2010 (Gómez-Baggethun et al. 2010; Nieto-Romero et al. 2014; Chaudhary et al. 2015; Hackbart et al. 2017), while more interdisciplinary research by applying socio-cultural valuation has emerged in the last decade (Chaudhary et al. 2015; Acharya et al. 2019; Martín-López et al. 2019). In fact, socio-cultural valuation studies became most prominent after the release of the TEEB report (TEEB 2010) and even more so after the release of the IPBES conceptual framework that specifically advocated for diverse values and valuation of nature (Díaz et al. 2015). Hence, one might argue that such international initiatives are a crucial driving force not only for compiling research and delivering guidance to policy-makers, but also for setting the research agenda. Moreover, the increase in socio-cultural valuation of protected areas goes along with the increasing inclusion of social science research for the management and conservation of protected areas (Palomo et al. 2014; Gruby et al. 2016; Ghoddousi et al. 2022).

Despite these recent developments, our results clearly show an overall bias towards monetary values and quantitative approaches (Fig. 5) that can be explained by two main reasons. First, the prominent pressure to provide economic justification for the conservation of protected areas (West et al. 2006; Balmford et al. 2009; do Val Simardi Beraldo Souza et al. 2018) might have encouraged researchers to focus on providing evidence of the economic importance of nature-based tourism in protected areas (Thur 2010; Gelcich et al. 2013; Aseres and Sira 2020). For example, Witt (2019) applied contingent valuation method to investigate tourists' willingness to pay for increased entrance fees in five Mexican protected areas. Soe Zin et al. (2019) estimated the annual recreational expenditure by tourists to elicit the economic value of cultural ecosystem services in Popa Mountain National Park (Myanmar). Second, the TEV framework in the research agenda fit with institutional economic structures based on market-based strategies (Gómez-Baggethun et al. 2010). For example, Chiou et al. (2016) applied contingent valuation to estimate tourists’ willingness to pay an entrance price in the Taitung Forest Park (Taiwan) to inform the Taitung Forest Park Administration Office about potential price making. The predominant economic logic in decision-making and management of protected areas might have prioritized the use of economic valuation and elicitation of economic values, often at the expense of the broad spectrum of other values. Corresponding with these results, the IPBES assessment highlights that the predominant economic decisions have generally fostered a narrow suite of instrumental values, particularly of those nature’s contributions to people that are traded in markets, obscuring those non-market instrumental, relational, and intrinsic values (IPBES 2022). Since it has been proved that economic valuation fails to represent the diverse ways by which people value nature (Jacobs et al. 2016, 2018), recent voices from IPBES have called for plural valuation (Pascual et al. 2017; IPBES 2022). Our results show that there is ample room for a stronger integration of methods from the social sciences and humanities. This integration would promote a more comprehensive understanding of the diverse values of nature, which might help to raise awareness of the importance of protected areas and to provide a greater diversity of arguments for their conservation and potential expansion.

#### Tourists’ valuation of nature in protected areas across regions

Research on tourists’ valuation of nature in protected areas has given major attention to protected areas in Asia, Africa, and Latin America and the Caribbean and focused less on protected areas in Europe, North America, and Oceania (Fig. 3b). This contrasts to some extent the findings of the IPBES assessment which concluded that most valuation studies have been conducted in the Americas, Asia, Oceania, and Europe (IPBES 2022).

When comparing the regional share of articles identified in the review with the relative distribution of protected areas across regions, we found that usually overstudied regions, such as Europe and North America, are comparatively understudied in tourists’ valuation of nature, whereas Asia, Africa, and Latin America and the Caribbean were overstudied (Fig. 3b). Moreover, our result implied a discrepancy between researched study areas and tourist interest in visiting protected areas considering the findings by Balmford et al. (2015). They estimated the number of visits to terrestrial protected areas based on the annual visit rate in each country from 1998 to 2007. While their results showed that Europe, North America, and Asia/Oceania are the regions of the world whose terrestrial protected areas received the highest visit rates, we found that these regions are relatively underrepresented in the research on tourists’ valuation of nature. We also found a low number of articles focusing on regions with high biodiversity, such as Africa, where the need for biodiversity conservation is high and nature-based tourism is essential for the livelihood security of local people (Sekar et al. 2014; Chung 2018).

Finally, our results suggested a bias towards the application of socio-cultural valuation in the Global North compared to the Global South (Fig. 3a). While studies in Africa applied socio-cultural valuation methods 20% less than the average application worldwide, studies in Europe applied them 9% more. The focus on economic valuation techniques in the Global South might be related to the fact that biodiversity conservation in protected areas tend to rely more strongly on revenues from nature-based tourism than those in the Global North*.* For example, in the Philippines, the National Ecotourism Strategy aims to integrate biodiversity conservation into ecotourism development and the funding mechanism for biodiversity conservation in protected areas (i.e., Integrated Protected Area Fund). The strategy is designed to ensure that visitor fees in protected areas are used for biodiversity conservation (Catibog-Sinha 2010). Yet, this bias could imply an underrepresentation of the non-economic values that tourists might hold on nature in protected areas in the Global South, which in turn could lead to an under-appreciation of these values in biodiversity conservation and protected area management. Here, we encourage the scientific community to elicit values of nature in those underrepresented regions by applying both economic and socio-cultural valuation. This is particularly important for protected areas in the Global South that attract a high number of tourists and where local people highly depend on the tourism sector for their livelihood security.

#### Tourists’ valuation of nature: What, who, and why?

Most literature on tourists’ valuation of nature focused, in addition to the protected area itself, on non-material nature's contributions to people and land-/seascapes (Supp. Material Table S7). The focus on these natural elements can explain why most of the research elicited recreational, direct use, and aesthetic values (in addition to economic values; Fig. 6). This echoes former reviews on cultural ecosystem services research which showed that recreation, tourism, and aesthetics are the most studied ecosystem services (Hernández-Morcillo et al. 2013; Milcu et al. 2013). Studies on land-/seascape perceptions are highly linked with eliciting aesthetic values, explaining the overrepresentation of both in the literature (Bogdan et al. 2019; Müller et al. 2019; Piñeiro-Corbeira et al. 2020). This result supports the findings by Hernández-Morcillo et al. (2013) who found that assessing landscapes retrospectively to identify the aesthetic values was a common method (Barthel et al. 2005; Vejre et al. 2010). In addition, reviews on ecosystem service research found that economic valuation techniques, particularly contingent valuation, are the most applied to measure recreational values (Hernández-Morcillo et al. 2013; Milcu et al. 2013). This can explain the bias towards non-material nature's contributions to people and land-/seascapes as well as recreational, direct use, and aesthetic values, in addition to the high number of economic values due to frequent application of economic valuation methods.

Regarding those who are targeted in the valuation exercises, only a third of the articles investigated the values of other social groups in addition to tourists (Fig. 4b). Recently, it has been argued that it is crucial to include multiple social groups to increase the success of conservation efforts in protected areas and mitigate potential conflicts among stakeholders (Kovács et al. 2015; Jacobs et al. 2016; Riechers et al. 2018). In addition, the consideration of the values of multiple stakeholders with different needs, interests, and worldviews is needed to represent the different voices in decision-making and management of protected areas (Jacobs et al. 2016, 2018). Here, we call for the inclusion of diverse stakeholder groups, such as tourists, local communities, and conservation managers, in future research on nature’s values in protected areas.

Finally, we found that the main objective of most studies on tourists’ values was value-oriented, aiming to compare values of different natural elements (Saayman and Saayman 2017; Robles-Zavala and Chang Reynoso 2018; Witt 2019) or elicit specific values derived from the protected area (Samdin et al. 2013; Dagiliūtė et al. 2017) (Fig. 4c). The second most frequent goal was tourist-oriented, whereby researchers aimed to identify the socio-economic or cultural characteristics of tourists that influence their value(s) (Queiroz et al. 2014; Pickering et al. 2020). Conservation management was the third most frequent research goal, through which researchers aim to provide guidelines for the conservation, management, and planning of the protected area (Can and Alp 2012; Saayman and Saayman 2014; Daly et al. 2015; Karahalil et al. 2015). Finally, science-oriented articles focused on the comparison of valuation methods or the development of conceptual frameworks (Font 2000; Walpole et al. 2001; Rossi et al. 2020).

Recent empirical research conducted in several case studies in the Global South have proved that when nature valuation aims to guide action on conservation and sustainability (i.e., conservation-management-oriented), research did not only succeed to elicit different values (for which different valuation methods are required), but also promote sustainability and environmental justice (Zafra-Calvo et al. 2020). For example, in Xalapa (Mexico), plural valuation was embedded in participatory action research and led to promote collective management of the protected area with successful conservation outcomes (Zafra-Calvo et al. 2020). In contrast, in the Otún watershed (Colombia), research on plural valuation did not lead to the inclusion of different stakeholder views in management plans (Arias-Arévalo et al. 2017). Therefore, we suggest that, if the valuation exercise seeks to contribute to nature conservation management, future research needs to move from economic valuation methods that only consult stakeholders to participatory valuation methods that actively engage with different stakeholders through transdisciplinary research approaches.

### Methods as value-articulating institutions

Research on tourists’ values of nature in protected areas was skewed towards quantitative and monetary data collection and consequently, most data were analyzed quantitatively (Fig. 5). Moreover, economic valuation focused particularly on eliciting economic (TEEB) and TEV values (Fig. 6). In addition, our results show that the choice of the valuation method determined the value type elicited (Fig. 8). This finding supports recent empirical evidence which demonstrated that valuation methods act as value-articulating institutions (Martín-López et al. 2014; Jacobs et al. 2018). This is consistent with previous theoretical debates which argue that valuation methods are not neutral and valuation itself acts as a value-articulating institution (Vatn and Bromley 1994; Vatn 2005). In fact, the choice of the method is as important as the output itself because methods do not simply ‘elicit’, but also ‘create’ values (Gómez-Baggethun and Ruiz-Pérez 2011; Martín-López et al. 2014). Therefore, to recognize the broad scope of values of nature in protected areas, we need to implement plural valuation, which aims to elicit diverse values and accounts for different worldviews by including diverse stakeholder groups, social–ecological characteristics, valuation methods, and power relations (Jacobs et al. 2016, 2020; Arias-Arévalo et al. 2017; Pascual et al. 2017). Although plural valuation might be seen as a complex and resource-consuming approach, Jacobs et al. (2018) found that the elicitation of diverse values of nature could be achieved by combining methods that do not necessarily require a higher cost. This review shows that socio-cultural valuation techniques elicited all value types, whereas economic valuation techniques elicited a much narrower spectrum of values (Fig. 6). Moreover, the IPBES assessment regarding the diverse conceptualization of diverse values of nature and its benefits calls for the use of multiple complementary methods ‘to make a wider diversity of values visible, while improving the quality and legitimacy of the information generated to support decisions about nature’ (IPBES 2022, p. 5).

Furthermore, social and ecological contexts of protected areas are very diverse and there is no one-size-fits-all valuation technique. Hence, the choice of valuation methods needs to be both context-specific and complementary to articulate the diverse values of nature (IPBES 2022). Likewise, the selection of the valuation framework should represent the social and ecological realities, while enabling the visibility of the diverse values of nature in protected areas. Our results show that, albeit different valuation frameworks have evolved over the years (Fig. 2), the use of TEV has been dominant when eliciting tourists’ values of nature (Fig. 6). Although the values found in the literature fit under the umbrella of different valuation frameworks (i.e., TEV, TEEB, and IPBES), these frameworks are not mutually exclusive and simultaneously used in current scientific literature (Fig. 7). In fact, the release of TEEB (TEEB 2010) or IPBES (Díaz et al. 2015) did not eclipse TEV, resulting in the use of multiple valuation frameworks today.

### Implications for policy-making and management of protected areas

The broad spectrum of tourists’ values of nature in protected areas requires the use of a variety of valuation techniques, and thus, the application of different methods may lead to several challenges when it comes to informing management decisions and policies (IPBES 2022). Furthermore, challenges might emerge when comparing results obtained through different valuation frameworks since their approaches, disciplines, and principles differ. Yet, these differences can precisely contribute to informing different aspects of protected area management. For example, while the TEV framework relies on economic values (and instrumental values) and can inform decisions related with market-based institutions (e.g., Payments for Ecosystem Services, entrance fee to visit a protected area), the emphasis on the relational values by the IPBES framework can inform decisions that nurture collective stewardship and design community-based management.

Despite the high potential to use the values of nature to inform management of protected areas, we found that only 34 articles aimed to provide recommendations for management or policy-making. In addition to the small share of research aiming to inform management and policy-making, the IPBES assessment regarding the diverse conceptualization of diverse values of nature and its benefits found that the great majority of peer reviewed studies on nature valuation does not seem to successfully impact real world decision-making (IPBES 2022).

Eliciting diverse values of nature does not seem to be enough to foster the uptake of valuation results in management and policy-making. Instead, Zafra-Calvo et al. (2020) found that valuation exercises can impact management decisions when the valuation process is embedded in transdisciplinary research. This finding corresponds the recent policy messages of the IPBES assessment which conclude that the uptake of valuation seems to be fostered by inclusive bottom-up processes where valuation knowledge is co-produced with multiple stakeholders (including decision-makers) through the application of participatory and deliberative methods (IPBES 2022).

Based on the findings of this review, we propose two main actions to foster the incorporation of values of nature in the management of protected areas. First, we propose to apply different and complementary valuation methods that represent the diverse ways by which tourists and other stakeholders relate with nature. To apply complementary methods, there is a need for building capacities and skills, particularly in developing countries (IPBES 2022). Since the research on tourists’ valuation of nature in developing regions is highly biased towards economic valuation, we suggest to build capacities and skills by applying socio-cultural valuation methods and mixed approaches to leverage the diverse values of nature into protected area management.

Second, we recommend to remove barriers that hinder the acknowledgement and expression of diverse values of nature in protected areas and to promote opportunities to do so (IPBES 2022). This might include, among others, an institutional (re-)orientation that moves towards nurturing and legitimizing worldviews and values of diverse types of tourists and other stakeholders. Here, we argue that IPBES acts as a key institution promoting the recognition and legitimation of multiple stakeholders’ worldviews and values. To do so, there is the need to create informative, participatory, and empowering spaces to express, negotiate, and deliberate diverse values by various stakeholders.

### Priorities for future research on tourists’ valuation of nature in protected areas

This systematic review was undertaken to present the status of empirical research on tourists’ values of nature in protected areas over time and across regions. As a result, we identified major research gaps and, accordingly, we suggest three priorities for the future research agenda:

1. Reduce regional bias: Our results show a bias towards certain regions and only marginal information on others (Fig. 3). Hence, we need scientific knowledge regarding values of protected areas in regions and countries that are currently underrepresented or not represented at all and continuous research in regions and countries with high visitation rates and biodiversity hotspots.
2. Diversify methods and stakeholders: Most research on eliciting values focused on quantitative and monetary approaches (Fig. 5), which are limiting the elicitation of some value types. Therefore, we advocate for the diversification of the toolset of methods by applying socio-cultural valuation techniques and mixed approaches that can provide insights into tourists’ non-monetary values of nature in protected areas. In addition, when the valuation exercise seeks to inform conservation and sustainable tourism in protected areas, it is necessary to account for the values of not just far-distant societies, but also local stakeholders, and to broaden the toolset towards participatory methods to engage different stakeholders and their diverse needs, interests, and worldviews (Jacobs et al. 2020; Zafra-Calvo et al. 2020). The engagement of diverse types of tourists and other stakeholders also requires addressing power relations among stakeholders (IPBES 2022).
3. Foster value pluralism: For a more comprehensive understanding on the diverse values that tourists and other stakeholder groups assign to nature in protected areas, research on value pluralism is a necessity. Recent developments, such as the IPBES valuation framework, have promoted the discussion of value pluralism in mainstream sustainability research. Our review, however, suggests that the uptake of value pluralism has been rather slow. Therefore, future research should be driven by new generations of researchers who are better trained in inter- and transdisciplinary approaches to overcome the disciplinary mindset in academia, acknowledge and research value pluralism, and allow the inclusion of diverse stakeholders.

## Conclusion

Valuation methods and frameworks used to elicit and understand tourists’ values of nature in protected areas have diversified in recent years. Considering their role as value-articulating institutions, we recommend that future research applies non-monetary and in-depth qualitative valuation methods, and conducts plural valuation to account for diverse stakeholders. This is essential for eliciting intrinsic, instrumental, and relational values, with the aim of fostering the conservation of protected areas and associated societal benefits.

## Supplementary Information

Below is the link to the electronic supplementary material.

Supplementary file1 (PDF 1845 KB)
Supplementary material S1 Prisma checklist (source: own elaboration based on Page et al. 2021). Supplementary material S2 Review procedure (source: own elaboration based on Pullin and Stewart (2006) and Luederitz et al. (2016)). Supplementary material S3 Description of inclusion criteria. Supplementary material S4 List of analyzed articles. Supplementary material S5 Review categories. Supplementary material S6 Examples of coded articles. Supplementary material S7 Additional results incl. Table S10 with quotes from the articles identified in this literature review to represent Figure 7 (source: own elaboration)

## Footnotes

1 This review was not registered.

2 https://www.researchgate.net/.
