# Correction: Tourists’ valuation of nature in protected areas: A systematic review

**DOI:** 10.1007/s13280-024-02123-3

**Published:** 2025-01-30

**Authors:** Milena Gross, Jasmine Pearson, Ugo Arbieu, Maraja Riechers, Simon Thomsen, Berta Martín-López

**Affiliations:** 1 Social-Ecological Systems Institute, Faculty of Sustainability Science, Leuphana University, Lüneburg, Universitätsallee 1, 21335 Lüneburg, Germany; 2 Senckenberg Biodiversity and Climate Research Centre, Frankfurt am Main, Georg-Voigt-Straße 14, 60325 Frankfurt am Main, Germany; 3 Smithsonian Conservation Biology Institute, National Zoological Park, 1500 Remount Road, Front Royal, VA 22630 USA; 4 Laboratoire d’Ecologie Systématique et Evolution, IDEEV, Université Paris-Saclay, Bât. 680 – 12, Route 128, 91190 Gif-sur-Yvette, France; 5 Institute of Ecology, Faculty of Sustainability Science, Leuphana University Lüneburg, Universitätsallee 1, 21335 Lüneburg, Germany

**Correction to: Ambio 2023, 52:1065–1084.** 10.1007/s13280-023-01845-0

In the original published article, authors have identified a mismatch between the main text, figures, and the provided data set. While the figures are correct and align with the data set whereas the main text contains incorrect information.

The sentence “Terrestrial ecosystems were more studied (n = 139; 70%) than marine (n = 61; 30%) and freshwater ecosystems (n = 46; 19%) (Fig. 4a)” appearing in the section **Ecological and social characteristics of case studies** should have been “Terrestrial ecosystems were more studied (n = 134; 88%%) than marine (n = 61; 40%) and freshwater ecosystems (n = 46; 30%) (Fig. 4a)”.

And the sentence “Most articles collected quantitative data (n = 114; 75% of all articles), followed by qualitative data (n = 24; 17%) and both data types (n = 5; 5%) (Fig. 5a)” appearing in the sub section *Data collection and analysis* should have been “Most articles collected quantitative data (n = 114; 75% of all articles), followed by qualitative data (n = 26; 17%) and both data types (n = 8; 5%) (Fig. 5a).”

The original article has been corrected.

