# Supplementary material for: Tourists’ valuation of nature in protected areas: A systematic review
Source: Ambio. 2023 Apr 18;52(6):1065–84. doi: 10.1007/s13280-023-01845-0 (PMC10160295; doi:10.1007/s13280-023-01845-0)
Supplement: Supplementary file 1 — Supplementary file1 (PDF 1845 KB) Supplementary material S1 Prisma checklist (source: own elaboration based on Page et al. 2021). Supplementary material S2 Review procedure (source: own elaboration based on Pullin and Stewart (2006) and Luederitz et al. (2016)). Supplementary material S3 Description of inclusion criteria. Supplementary material S4 List of analyzed articles. Supplementary material S5 Review categories. Supplementary material S6 Examples of coded articles. Supplementary material S7 Additional results incl. Table S10 with quotes from the articles identified in this literature review to represent Figure 7 (source: own elaboration) [file 13280_2023_1845_MOESM1_ESM.pdf]

Ambio

Electronic Supplementary Material

This supplementary information has not been peer-reviewed.

Title: ***Tourist's valuation of nature in protected areas – a systematic review***

Milena Gross, Jasmine Pearson, Ugo Arbieu, Maraja Riechers, Simon Thomson, Berta Martín-López

Supplementary Material S1

Table S1: Prisma checklists for systematic literature reviews based on Page et al. (2021)

Section and Topic	Item #	Checklist item	Location where item is reported
**TITLE**			
**Title**	**1**	Identify the report as a systematic review.	Title, Abstract, Methods
**ABSTRACT**			
**Abstract**	**2**	See the PRISMA 2020 for Abstracts checklist.	Drafting of abstract was guided by criteria and considered, if limited numbers of words allowed to do so.
**INTRODUCTION**			
**Rationale**	**3**	Describe the rationale for the review in the context of existing knowledge.	Introduction
**Objectives**	**4**	Provide an explicit statement of the objective(s) or question(s) the review addresses.	Introduction
**METHODS**			
**Eligibility criteria**	5	Specify the inclusion and exclusion criteria for the review and how studies were grouped for the syntheses.	Methods, Supp. Material S2, Supp. Material S3
**Information sources**	6	Specify all databases, registers, websites, organizations, reference lists and other sources searched or consulted to identify studies. Specify the date when each source was last searched or consulted.	Methods, Supp. Material S2

**Search strategy**	7	Present the full search strategies for all databases, registers and websites, including any filters and limits used.	Methods, Supp. Material S2
**Selection process**	8	Specify the methods used to decide whether a study met the inclusion criteria of the review, including how many reviewers screened each record and each report retrieved, whether they worked independently, and if applicable, details of automation tools used in the process.	Methods, Supp. Material S2
**Data collection process**	9	Specify the methods used to collect data from reports, including how many reviewers collected data from each report, whether they worked independently, any processes for obtaining or confirming data from study investigators, and if applicable, details of automation tools used in the process.	Methods
**Data items**	10a	List and define all outcomes for which data were sought. Specify whether all results that were compatible with each outcome domain in each study were sought (e.g., for all measures, time points, analyses), and if not, the methods used to decide which results to collect.	NA
	10b	List and define all other variables for which data were sought (e.g., participant and intervention characteristics, funding sources). Describe any assumptions made about any missing or unclear information.	NA
**Study risk of bias assessment**	11	Specify the methods used to assess risk of bias in the included studies, including details of the tool(s) used, how many reviewers assessed each study and whether they worked independently, and if applicable, details of automation tools used in the process.	Methods
**Effect measures**	12	Specify for each outcome the effect measure(s) (e.g., risk ratio, mean difference) used in the synthesis or presentation of results.	NA
**Synthesis methods**	13a	Describe the processes used to decide which studies were eligible for each synthesis (e.g., tabulating the study intervention characteristics and comparing against the planned groups for each synthesis (item #5)).	All articles were eligible.
	13b	Describe any methods required to prepare the data for presentation or synthesis, such as handling of missing summary statistics, or data conversions.	Supp. Material S5, Supp. Material S6

	13c	Describe any methods used to tabulate or visually display results of individual studies and syntheses.	Methods
	13d	Describe any methods used to synthesize results and provide a rationale for the choice(s). If meta-analysis was performed, describe the model(s), method(s) to identify the presence and extent of statistical heterogeneity, and software package(s) used.	Methods
	13e	Describe any methods used to explore possible causes of heterogeneity among study results (e.g., subgroup analysis, meta-regression).	NA
	13f	Describe any sensitivity analyses conducted to assess robustness of the synthesized results.	NA
**Reporting bias assessment**	14	Describe any methods used to assess risk of bias due to missing results in a synthesis (arising from reporting biases).	NA
**Certainty assessment**	15	Describe any methods used to assess certainty (or confidence) in the body of evidence for an outcome.	NA
**RESULTS**			
**Study selection**	16a	Describe the results of the search and selection process, from the number of records identified in the search to the number of studies included in the review, ideally using a flow diagram.	Methods, Supp. Material S2
	16b	Cite studies that might appear to meet the inclusion criteria, but which were excluded, and explain why they were excluded.	Supp. Material S3
**Study characteristics**	17	Cite each included study and present its characteristics.	Supp. Material S4

**Risk of bias in studies**	18	Present assessments of risk of bias for each included study.	NA
**Results of individual studies**	19	For all outcomes, present, for each study: (a) summary statistics for each group (where appropriate) and (b) an effect estimate and its precision (e.g., confidence/credible interval), ideally using structured tables or plots.	NA
**Results of syntheses**	20a	For each synthesis, briefly summarize the characteristics and risk of bias among contributing studies.	Methods, Supp. Material S3
	20b	Present results of all statistical syntheses conducted. If meta-analysis was done, present for each the summary estimate and its precision (e.g., confidence/credible interval) and measures of statistical heterogeneity. If comparing groups, describe the direction of the effect.	Results, Figure 8
	20c	Present results of all investigations of possible causes of heterogeneity among study results.	NA
	20d	Present results of all sensitivity analyses conducted to assess the robustness of the synthesized results.	NA
**Reporting biases**	21	Present assessments of risk of bias due to missing results (arising from reporting biases) for each synthesis assessed.	NA
**Certainty of evidence**	22	Present assessments of certainty (or confidence) in the body of evidence for each outcome assessed.	NA
**DISCUSSION**			
**Discussion**	23a	Provide a general interpretation of the results in the context of other evidence.	Discussion
	23b	Discuss any limitations of the evidence included in the review.	Methods, Supp. Material S3

	23c	Discuss any limitations of the review processes used.	Methods, Supp. Material S3
	23d	Discuss implications of the results for practice, policy, and future research.	Discussion
**OTHER INFORMATION**			
**Registration and protocol**	24a	Provide registration information for the review, including register name and registration number, or state that the review was not registered.	Footnote in Methods
	24b	Indicate where the review protocol can be accessed, or state that a protocol was not prepared.	Supp. Material S2
	24c	Describe and explain any amendments to information provided at registration or in the protocol.	NA
**Support**	25	Describe sources of financial or non-financial support for the review, and the role of the funders or sponsors in the review.	Acknowledgement
**Competing interests**	26	Declare any competing interests of review authors.	Declaration of Competing Interest
**Availability of data, code and other materials**	27	Report which of the following are publicly available and where they can be found: template data collection forms; data extracted from included studies; data used for all analyses; analytic code; any other materials used in the review.	Publicly available on ResearchGate 10.13140/RG.2.2.29999.00161

Reference

Page, M.J., J.E. McKenzie, P.M. Bossuyt, I. Boutron, T.C. Hoffmann, C.D. Mulrow, L. Shamseer, J.M. Tetzlaff, et al. 2021. The PRISMA 2020 statement: an updated guideline for reporting systematic reviews. BMJ: n71. doi:10.1136/bmj.n71.

Supplementary Material S2

Table S2: Review procedure based on Luederitz et al. (2016) and Pullin and Stewart (2006)

Review steps	Procedure	Result
1. Data gathering	Database search on Scopus using the following search string with redundant search words for subject: tourists; object: value; focus of value: nature; study area: protected area.<br><br>(TITLE-ABS-KEY (touris* OR recreation* OR leisure OR travel* OR trip* OR journey*) AND TITLE-ABS-KEY (*valu*) AND TITLE-ABS-KEY ("protected area*" OR "protected landscape*" OR "biosphere reserve*" OR "nature reserve*" OR "natural monument*" OR "national park*" OR "natural park*" OR "biosphere area*" OR "conservation area*" OR "biosphere region*"))) AND (LIMIT-TO (DOCTYPE , "ar")) AND (EXCLUDE (SUBJAREA , "BIOC") OR EXCLUDE (SUBJAREA , "ENGI") OR EXCLUDE (SUBJAREA , "MEDI") OR EXCLUDE (SUBJAREA , "IMMU") OR EXCLUDE (SUBJAREA , "COMP") OR EXCLUDE (SUBJAREA , "PHYS") OR EXCLUDE (SUBJAREA , "CHEM") OR EXCLUDE (SUBJAREA , "PHAR") OR EXCLUDE (SUBJAREA , "VETE") OR EXCLUDE (SUBJAREA , "MATH") OR EXCLUDE (SUBJAREA , "NEUR") OR EXCLUDE (SUBJAREA , "CENG") OR EXCLUDE (SUBJAREA , "MATE")) AND (LIMIT-TO (LANGUAGE , "English")<br><br>TITLE-ABS-KEY indicates search word appearing in title, abstract, and/or keywords. LIMIT-TO indicates restrictions (to type of publication (ar: article), i.e., exclusion of, e.g., book chapter and conference proceedings) to avoid including the same empirical data more than once, and language (English)). EXCLUDE indicates exclusion (of subject areas here). We excluded articles associated with non-related Web of Science Categories (e.g., Engineering, Medicine, and Neuroscience) a priori. Search terms were grouped by the three sets subject, object, and study area. The Boolean operator 'OR' was added to separate search words within a set and the operator 'AND' to separate the four sets. The asterisk * was added to account for diverse prefixes and suffixes. By doing so, potentially relevant articles contain at least one word from each set (Pullin et al. 2013). Language of the articles was restricted to English. Only peer-reviewed articles were considered. Subject areas[1] that do not relate to the	Bibliographic information of 2526 potentially relevant articles available as of 23 June, 2021.

[1] BIOC: Biochemistry, Genetics and Molecular Biology; CENG: Chemical Engineering, CHEM: Chemistry; COMP: Computer Science; ENGI: Engineering; IMMU: Immunology and Microbiology; MATH: Mathematics; MEDI: Medicine; NEUR: Neuroscience; PHAR: Pharmacology, Toxicology and Pharmaceutics; PHYS: Physics and Astronomy; VETE: Veterinary.

	focus of the study were excluded a priori. No date restrictions were applied.	
2. Data screening	Based on an iterative process, numerous articles were screened to define inclusion criteria.	Definition of inclusion criteria.
3. Data cleaning	Screening of title and abstract of 2526 articles, guided by the following five inclusion criteria:<br>1. Subject: Are *tourists* sampled?<br>2. Object: Does the article elicit tourists' (contextual) *value(s)* through a valuation method?<br>3. Focus of value: Is the (contextual) value of (any element of) *nature* collected?<br>4. Study area: Was the study area a *protected area*?<br>5. Case study: Does the article provide *empirical data* on the research topic?<br>Inclusion criteria are described in detail in Supp. Material S3.<br>If the abstract was missing or information insufficient, articles were accepted for full text screening.	846 potentially relevant articles were identified.
4. Data scoping	Download of all articles classified as potentially relevant. Three articles were not accessible, although articles were requested through email from at least one author and/or ResearchGate. (A reminder was sent as well.)	Download of 843 potentially relevant studies.
5. Article classification	Screening of full text of potentially relevant articles according to inclusion criteria.	152 articles that contain relevant information serving the overall objective and the three research questions (Supp. Material S4).
7. Article review	Analysis of relevant articles using 18 review categories (Supp. Material S5).	Coherent dataset of 152 articles with 18 review categories each. See Supp. Material S6 for examples of coded articles.
8. Data analysis	Analysis of all relevant data using RStudio.	See Results.

References

Luederitz, C., M. Meyer, D.J. Abson, F. Gralla, D.J. Lang, A.-L. Rau, and H. von Wehrden. 2016. Systematic student-driven literature reviews in sustainability science – an effective way to merge research and teaching. *Journal of Cleaner Production* 119: 229–235. doi:10.1016/j.jclepro.2016.02.005.

Pullin, A.S., and G.B. Stewart. 2006. Guidelines for Systematic Review in Conservation and Environmental Management. *Conservation Biology* 20: 1647–1656. doi:10.1111/j.1523-1739.2006.00485.x.

Pullin, A.S., M. Bangpan, S. Dalrymple, K. Dickson, N.R. Haddaway, J.R. Healey, H. Hauari, N. Hockley, et al. 2013. Human well-being impacts of terrestrial protected areas. *Environmental Evidence* 2: 19. doi:10.1186/2047-2382-2-19.

Supplementary Material S3

Description of inclusion criteria

1. Inclusion criterion: case study

Case study: Does the article provide *empirical data* on the research topic?

Search string terms: We intentionally did not include any search term related to case studies because of the selection rule specified below.

We only included studies that provide empirical data on the research topic, whereas we excluded, for example, purely theoretical and conceptual articles, systematic literature reviews, meta-analysis, and gray literature to avoid including the same empirical data more than once and to assure scientific quality of the reviewed articles. Hence, we did not include any (redundant) search terms related to the inclusion criterion case study in the search string because we did not want to restrict the search of potentially relevant articles due to missing search terms. This inclusion criterion is rather a matter of reading and interpreting title, abstract, and main text. Case study and empirical study, for example, were indicator words for this inclusion criterion.

2. Inclusion criterion: value

Object: Does the article elicit tourists' (contextual) *value(s)* through a valuation method?

Search string terms: *valu* - positively identifiable words in title, abstract, and key words, e.g., (to) value, value, valuation; singular and plural versions and different tenses of some search terms.

There are many definitions of and uses for the term value found in the literature (Kenter et al. 2015). To be consistent in this review, we included contextual values only according to the definition stated in Kenter et al. (2015, p. 92), that is, "opinions about the importance or worth of something". Therefore, we developed the following four selection rules:

1. Articles on, e.g., transcendental values, value-belief-norm, new environmental paradigm, pro-environmental behavior and attitude, and those that used buzzwords such as values of nature-based tourism or ecotourism and proxies such as number of tourists or financial benefit did not meet our inclusion criterion value and hence, were excluded. Because of this selection rule, the following article was excluded, for example, Vásquez Lavín et al. (2016).
2. We only included articles in which the authors clearly stated what they referred to as value and/ or applied a valuation method, i.e., valuation exercise. We did not interpret additional data nor extract data beyond the author's definition. For example, Abu Bakar et al. (2016), whose ranking / rating exercise can be used to elicit contextual values but was not stated by the authors as a valuation method, was excluded from the final set of relevant articles.

3. We only included articles, which displayed the results of tourists' valuation exercise. Because of this selection rule the following article was excluded, for example, Heslinga et al. (2017).
4. Articles were excluded if they were found in the Scopus search due to the appearance of the terms 'valuable' or 'evaluation' in title, abstract and/or key words but did not refer to a contextual value or valuation method / exercise. Because of this selection rule the following articles were excluded, for example, Kulczycki and Halpenny (2014) and Mills et al. (1980).

Our search string term for the inclusion criterion value might have overlooked research on tourists' values of nature in protected areas because we decided to not use synonyms. Two reasons explain this decision. First, since the aim of this research was to explore how the scientific literature has approached nature valuation, we deliberately targeted those articles that had used the concept of value / valuation. Former systematic reviews have applied a similar approach by narrowing their search string to value / valuation and not adding synonyms for valuation (e.g., Acharya et al. 2019). Second, the use of other terms in the search that could potentially refer to value, such as perceptions, can lead to many articles that do not focus on nature valuation but on related yet different concepts such as beliefs, attitudes, and norms.

3. Inclusion criterion: nature

Focus of value: Is the (contextual) value of (any element of) *nature* elicited?

Search string terms: We intentionally did not include any search term related to natural elements because they are diverse, and we did not want to restrict the search due to potentially missing search terms. We specified the following three selection rules:

1. We referred to nature as any abiotic, biotic, tangible and intangible element of nature and any nature-mediated element, e.g., landscape, animal, species composition, sound, ecosystem, clouds, and rocks, scuba-diving, and Ecosystem Services / Nature's Contributions to People.
2. Articles on management and conservation plans and/or scenarios that considered elements of nature in the valuation exercise met the inclusion criterion nature (e.g. Iranah et al. 2018).
3. Articles on general management and/or conservation plans and/or scenarios, that is, they did not target natural elements specifically but only non-natural management options of protected areas (e.g., maintenance of trails, educational programs, facilities, and number of benches) through the valuation exercise (mainly, e.g., choice experiments and willingness to pay exercises), did not meet the inclusion criteria nature and hence, were excluded.

Table S3 provides an exemplary overview with arguments for inclusion or exclusion of valuation methods to elicit the value of natural and non-natural elements of nature.

Table S3: Overview of inclusion and exclusion criteria for the inclusion criterion nature

Valuation method and object of value	Argument for inclusion and exclusion	Natural element	Reference
Willingness to pay for management and conservation operation	Exclusion. We don't know whether the management and conservation operation refer to (a) natural element(s) because the authors did not specify.	NA	Abu Bakar et al. (2016)
Willingness to pay for ecotourism development	Exclusion. The authors did not specify the (potential) element of nature.	NA	Chen and Jim (2012)
Willingness to pay for conservation of forest	Inclusion. The focus of conservation is the natural element forest.	Forest	Iranah et al. (2018)
Willingness to pay for invasive species management	Inclusion. Invasive species are an element of nature qua definition.	Invasive species	García-Llorente et al. (2011)
Willingness to pay for entering national park	Inclusion. The entrance fee provides access to the national park.	National Park	Mathieu et al. (2003)
Choice experiment:<br>1. Area (size of the area protected, km^2)<br>2. Ethiopian wolf population (number of wolves)<br>3. Interpretive signs and improved walking trails<br>4. Access (access to the wolf habitat)<br>5. Fee (park entry fee per person per day in ETB)	Inclusion. The protected area and the Ethiopian wolf are, for example, natural elements qua definition. Interpretive signs and improved walking trails are considered as non-natural elements.	Protected area, wolf	Estifanos et al. (2021)
Choice experiment:<br>1. Level of encounters with other visitors<br>2. Ecological condition of official Trail<br>3. Number of people with access to trails<br>4. Trail management	Exclusion. Only non-natural elements are valued.	NA	Cahill et al. (2007)

4. Inclusion criterion: study area

Study area: Is the study area a *protected area*?

Search string terms: "protected area*" OR "protected landscape*" OR "biosphere reserve*" OR "nature reserve*" OR "natural monument*" OR "national park*" OR "natural park*" OR "biosphere area*" OR "conservation area*" OR "biosphere region*" - positively identifiable words in title, abstract, and key words: singular and plural versions of the search terms

The definition of the term protected area was broad and context-specific because it was based on any sub-national, national and/or international (e.g., IUCN categories of protected areas)

legal definition of a *natural* area that is under protection qua law. Furthermore, we specified three selection rules:

1. Articles that scrutinized tourists' value of nature in a protected area, nature of a network of protected areas, and/or of multiple protected areas met the inclusion criterion study area. Because of this selection rule the following articles were excluded, for example, Pereira et al. (2019) and Vianna et al. (2012).
2. Articles on a hypothetical protected area or a study area adjacent to a protected area did not meet the inclusion criterion study area. Because of this selection rule the following article was excluded, for example, Vianna et al. (2018).
3. If the study area included both protected and non-protected areas but the results were not separated for them, the article did not meet the inclusion criterion study area.

5. Inclusion criterion: tourist

Subject: Are *tourists* sampled?

Search string terms: touris* OR recreation* OR leisure OR travel* OR trip* OR journey* - positively identifiable words in title, abstract, and key words, e.g., tourist, tourism, touristic, recreation, recreational; singular and plural versions of some search terms.

Originally, we wanted to develop a precise definition of the inclusion criterion tourist, e.g., based on the distance travelled from home to the protected area. However, after screening potentially relevant articles to develop such a definition (see review step 2 data screening in Table S2), we found that the majority of the reviewed articles did not provide a precise definition and thus, it was not possible to develop a consistent definition and apply it across all potentially relevant articles. In fact, we also assumed the definition of tourist depends on the local context. Therefore, articles generally met the inclusion criterion 'tourist', if authors used the term tourist to describe their target group or one of their investigated target groups, e.g., local, non-local, national, and international tourists.

While screening the relevant articles, we learnt authors used synonyms for the term tourist, such as visitors, travelers, foreigners, and non-locals, to describe their target group. Therefore, we developed, first, our working definition (see (1) below) of the term tourist and, second, inclusion rules to account for synonyms of the term tourist aligned with our working definition (see (2) below). Finally, we could not apply our working definition and inclusion rules to two valuation methods that were used in the articles found in the Scopus search, that is, zonal travel cost and public method based on social media and photo-based data. At the same time, we did not want to neglect this information on values, as the rationale inherent to the valuation method did not allow us to apply our working definition and selection rules. Thus, we defined specific selection rules for these two valuation methods (see (3) below).

(1) Working definition of the term tourist:

We defined a tourist as any person who travels to and visits the protected area in their free time for hours to days, that is, day and overnight tourists. We did not apply a minimum travel distance to classify a person as a tourist because this information was also not stated by the majority of the articles (with two exceptions see (3) below). People who are present in or in the surroundings of the protected area in the context of their daily life (e.g., (indigenous)

residents, local people, local community, visitors from the region) and job duties (e.g., park managers and decision-makers) or we investigated through a household survey in or in the surroundings of the protected area were not considered tourists. Hence, articles that sampled these target groups only were excluded.

(2) Selection rules to account for synonyms of the term tourist:

While screening the articles, we encountered potential synonyms for the term tourist, e.g., visitor, traveler, foreigner, and non-local. We did not want to bias our sampling process by just including articles that strictly use the term tourist, as we would have excluded many relevant articles that use synonyms. Thus, we interpreted the synonyms based on our working definition. To make consistent decisions, we developed standardized selection rules to account for the synonyms. The terms traveler, foreigner, and non-local, for example, generally met our working definition. The term visitor seemed to be more challenging because it is less specific than the term tourist: All tourists are visitors but not all visitors are tourists. The term visitor can include local people who visit the protected area, for example, but local people[2] do not meet our working definition of the term tourist. Therefore, the following six selection rules were developed and applied to avoid extracting data from mixed samples, i.e., mainly local people and tourists.

1. If authors used the terms tourists and visitors as synonyms, the article met the inclusion criterion.
2. If authors used the term visitors but not tourists and did not specify the geographical origin of the visitors, the article did not meet the inclusion criterion tourist and hence, was excluded to avoid having a mixed sample that included, e.g., local and foreign visitors. Because of this selection rule, the following articles were excluded, for example, Becker et al. (2005) and Englin et al. (2006).
3. If authors used the term visitor to describe a target group and distinguished it from local people as a separate target group, these visitors were considered tourists and hence, the article met the inclusion criterion tourist.
4. If the authors distinguished between local and foreign *visitors*, foreign visitors only met the inclusion criterion tourist.
5. If the authors distinguished between any of the three groups local/nearby(in-state), domestic/national/long-distance (out-of-state), and foreign/international *visitors*, local visitors did not but the other type of visitors met the inclusion criterion tourist, e.g., Richardson et al. (2006).
6. If the authors distinguished between the terms *non-visitor* and a *visitor* who visits the protected area in their leisure time, non-visitors did not but visitors who visited the protected area in their leisure time met the inclusion criterion tourist.

(3) Definition of the term tourist regarding specific methods:

We defined specific selection rules for zonal travel cost method as well as social media and photo platform-based valuation methods. Articles applying these valuation methods usually use the term visitor to describe their samples.

[2] We decided to differentiate between the target groups residents, locals or local people and target groups tourists / travelers / foreigners because authors argue that these groups value natural elements differently due to their different place attachments with the respective protected area (Larkin and Beier 2014; Mrotek et al. 2019). Thus, we only considered articles to meet this first inclusion criterion tourist if articles displayed tourists' values distinctively from the other target groups. By doing so, we avoided to extract data from mixed samples.

- Zonal travel cost method: If the authors considered ‘visitors’ who travelled a minimum distance of 100km to the protected area and display their travel cost, the article met the inclusion criterion. Because of this selection rule the following article was excluded, for example, Becker et al. (2005).
- Public method based on social media and photo-based data: Articles analyzed the social media content of users and (other) photo platforms, such as Flickr.com, to elicit visitor’s values to protected areas. Authors did not personally consult the users or photographers. Hence, critical information on the role they took on in the protected area was missing. Thus, values investigated for different target groups such as locals and tourists can hardly be distinguished in the valuation method of public method (e.g., Martínez Pastur et al. 2016). Nevertheless, we decided that such articles met the inclusion criterion, as the method used did not allow the authors to provide this information. We are aware that values of locals might have been included in our literature review and see this decision as a limitation of our review. However, we recognized the value of this rather new valuation method, e.g., to elicit values from visitors based on a large database that is easily and globally accessible and wanted to demonstrate how the scientific community has been applying this method in the scientific field of tourists’ values of nature in protected areas.

References

Abu Bakar, N.A., A. Radam, Z. Samdin, and M.R. Yacob. 2016. Willingness to pay in Kubah National Park and Matang Wildlife Centre: a contingent valuation method. *International Journal of Business and Society* 17. doi:10.33736/ijbs.517.2016.

Acharya, R.P., T. Maraseni, and G. Cockfield. 2019. Global trend of forest ecosystem services valuation – An analysis of publications. *Ecosystem Services* 39: 100979. doi:10.1016/j.ecoser.2019.100979.

Becker, N., M. Inbar, O. Bahat, Y. Choresh, G. Ben-Noon, and O. Yaffe. 2005. Estimating the economic value of viewing griffon vultures Gyps fulvus : a Travel Cost Model study at Gamla Nature Reserve, Israel. *Oryx* 39: 429. doi:10.1017/S0030605305001122.

Cahill, K.L., J.L. Marion, and S.R. Lawson. 2007. Enhancing the Interpretation of Stated Choice Analysis Through the Application of a Verbal Protocol Assessment. *Journal of Leisure Research* 39: 201–221. doi:10.1080/00222216.2007.11950105.

Chen, W.Y., and C.Y. Jim. 2012. Contingent valuation of ecotourism development in country parks in the urban shadow. *International Journal of Sustainable Development & World Ecology* 19: 44–53. doi:10.1080/13504509.2011.588727.

Englin, J.E., J.M. McDonald, and K. Moeltner. 2006. Valuing ancient forest ecosystems: An analysis of backcountry hiking in Jasper National Park. *Ecological Economics* 57: 665–678. doi:10.1016/j.ecolecon.2005.05.022.

Estifanos, T., M. Polyakov, R. Pandit, A. Hailu, and M. Burton. 2021. What are tourists willing to pay for securing the survival of a flagship species? The case of protection of the Ethiopian wolf. *Tourism Economics* 27: 45–69. doi:10.1177/1354816619880430.

García-Llorente, M., B. Martín-López, P.A.L.D. Nunes, J. A. González, P. Alcorlo, and C. Montes. 2011. Analyzing the Social Factors That Influence Willingness to Pay for Invasive Alien Species Management Under Two Different Strategies: Eradication and Prevention. *Environmental Management* 48: 418–435. doi:10.1007/s00267-011-9646-z.

Heslinga, J.H., P. Groote, and F. Vanclay. 2017. Using a social-ecological systems perspective to understand tourism and landscape interactions in coastal areas. *Journal of Tourism Futures* 3: 23–38. doi:10.1108/JTF-10-2015-0047.

Iranah, P., P. Lal, B.T. Wolde, and P. Burli. 2018. Valuing visitor access to forested areas and exploring willingness to pay for forest conservation and restoration finance: The case of small island developing state of Mauritius. *Journal of Environmental Management* 223: 868–877. doi:10.1016/j.jenvman.2018.07.008.

Kenter, J.O., L. O'Brien, N. Hockley, N. Ravenscroft, I. Fazey, K.N. Irvine, M.S. Reed, M. Christie, et al. 2015. What are shared and social values of ecosystems? *Ecological Economics* 111: 86–99. doi:10.1016/j.ecolecon.2015.01.006.

Kulczycki, C., and E.A. Halpenny. 2014. Sport cycling tourists' setting preferences, appraisals and attachments. *Journal of Sport & Tourism* 19: 169–197. doi:10.1080/14775085.2015.1070741.

Larkin, A.M., and C.M. Beier. 2014. Wilderness perceptions versus management reality in the Adirondack Park, USA. *Landscape and Urban Planning* 130: 1–13. doi:10.1016/j.landurbplan.2014.06.003.

Martínez Pastur, G., P.L. Peri, M.V. Lencinas, M. García-Llorente, and B. Martín-López. 2016. Spatial patterns of cultural ecosystem services provision in Southern Patagonia. *Landscape Ecology* 31: 383–399. doi:10.1007/s10980-015-0254-9.

Mathieu, L.F., I.H. Langford, and W. Kenyon. 2003. Valuing marine parks in a developing country: a case study of the Seychelles. *Environment and Development Economics* 8: 373–390. doi:10.1017/S1355770X0300196.

Mills, A.S., J.G. Massey, and H.M. Gregersen. 1980. Benefit-Cost Analysis of Voyageurs National Park. *Evaluation Review* 4: 715–738. doi:10.1177/0193841X8000400601.

Mrotek, A., C.B. Anderson, A.E. Valenzuela, L. Manak, A. Weber, P. Van Aert, M. Malizia, and E.A. Nielsen. 2019. An evaluation of local, national and international perceptions of benefits and threats to nature in Tierra del Fuego National Park (Patagonia, Argentina). *Environmental Conservation* 46: 326–333. doi:10.1017/S0376892919000250.

Pereira, L.S., D.M. de Carvalho, and L.S. da Cunha. 2019. Methodology for the Semi-quantitative Evaluation of Geoheritage Applied to Coastal Geotourism in João Pessoa (Paraíba, Northest Brazil). *Geoheritage* 11: 1941–1953. doi:10.1007/s12371-019-00417-7.

Richardson, R.B., J. Loomis, and S. Weiler. 2006. Recreation as a Spatial Good: Distance Effects on Changes in Recreation Visitation and Benefits. *Review of Regional Studies* 36. doi:10.52324/001c.8325.

Vásquez Lavín, F., S. Gelcich, X. Paz Lerdón, and F. Montealegre Bustos. 2016. The role of information in changing tourists behavioral preferences at the Humboldt penguin reserve in northern Chile. *Ocean & Coastal Management* 125: 63–69. doi:10.1016/j.ocecoaman.2016.03.003.

Vianna, G.M.S., M.G. Meekan, D.J. Pannell, S.P. Marsh, and J.J. Meeuwig. 2012. Socio-economic value and community benefits from shark-diving tourism in Palau: A sustainable use of reef shark populations. *Biological Conservation* 145: 267–277. doi:10.1016/j.biocon.2011.11.022.

Vianna, G.M.S., M.G. Meekan, A.A. Rogers, M.E. Kragt, J. M. Alin, and J.S. Zimmerhackel. 2018. Shark-diving tourism as a financing mechanism for shark conservation strategies in Malaysia. *Marine Policy* 94: 220–226. doi:10.1016/j.marpol.2018.05.008.

Supplementary Material S4

Table S4: List of the 152 articles analyzed in this literature review

Author(s)	Title	Year	Journal title	DOI
Affek A.N., Kowalska A.	Ecosystem potentials to provide services in the view of direct users	2017	Ecosystem Services	10.1016/j.ecoser.2017.06.017
Ågren J., Nyyssölä C., Stage J.	The potential for monopoly rents from Etosha National Park, Namibia	2003	South African Journal of Economic and Management Sciences	10.4102/sajems.v6i3.3300
Amirnejad H., Ataie Solout K.	Economic valuation of use values of environmental services in lar national park in Iran	2021	Journal of Agricultural Science and Technology	
Amirnejad H., Jahanifar K.	Comparison of contingent valuation and travel cost method in estimating the recreational values of a forest park	2018	Journal of Environmental Science and Management	
Anna Z., Saputra D.S.	Economic valuation of whale shark tourism in Cenderawasih Bay National Park, Papua, Indonesia	2017	Biodiversitas	10.13057/biodiv/d180321
Antoušková M.	Economic value of recreation - Determinants influencing the willingness to pay in natural region with low-intensity agriculture	2012	Agris On-line Papers in Economics and Informatics	
Antoušková M.	Comparison of take-it-or-leave-it and take-it-or-leave-it with follow-up elicitation formats - case study of Czech national parks	2012	Acta Universitatis Agriculturae et Silviculturae Mendelianae Brunensis	10.11118/actaun201260070019
Apps K., Dimmock K., Lloyd D.J., Huveneers C.	What values do tourists place on a marine protected area? White shark cage-dive tourists and the Neptune Islands	2019	Tourism in Marine Environments	10.3727/154427319X15567690 274868
Asafu-Adjaye J., Tapsuwan S.	A contingent valuation study of scuba diving benefits: Case study in Mu Ko Similan Marine National Park, Thailand	2008	Tourism Management	10.1016/j.tourman.2008.02.005

Author(s)	Title	Year	Journal title	DOI
Aseres S.A., Sira R.K.	Estimating visitors' willingness to pay for a conservation fund: sustainable financing approach in protected areas in Ethiopia	2020	Heliyon	10.1016/j.heliyon.2020.e04500
Badola R., Hussain S.A., Mishra B.K., Konthoujam B., Thapliyal S., Dhakate P.M.	An assessment of ecosystem services of Corbett Tiger Reserve, India	2010	Environmentalist	10.1007/s10669-010-9278-5
Baral N., Hazen H., Thapa B.	Visitor perceptions of World Heritage value at Sagarmatha (Mt. Everest) National Park, Nepal	2017	Journal of Sustainable Tourism	10.1080/09669582.2017.1291647
Baral N., Kaul S., Heinen J.T., Ale S.B.	Estimating the value of the World Heritage Site designation: a case study from Sagarmatha (Mount Everest) National Park, Nepal	2017	Journal of Sustainable Tourism	10.1080/09669582.2017.1310866
Barry S.J.	Using social media to discover public values, interests, and perceptions about cattle grazing on park lands	2014	Environmental Management	10.1007/s00267-013-0216-4
Bennett J., Gillespie R., Powell R., Chalmers L.	The economic value and regional economic impact of national parks	1996	Australian Journal of Environmental Management	10.1080/14486563.1996.10648360
Bhat M.Y., Bhatt M.S.	Economic valuation of biodiversity in South Asia: The case of Dachigam National Park in Jammu and Kashmir (India)	2019	Asia and the Pacific Policy Studies	10.1002/app5.266
Bhattarai B.R., Morgan D., Wright W.	Equitable sharing of benefits from tiger conservation: Beneficiaries' willingness to pay to offset the costs of tiger conservation	2021	Journal of Environmental Management	10.1016/j.jenvman.2021.112018
Bogdan S.M., Stupariu I., Andra-Topârceanu A., Năstase I.I.	Mapping social values for cultural ecosystem services in a mountain landscape in the Romanian Carpathians	2019	Carpathian Journal of Earth and Environmental Sciences	10.26471/cjees/2019/014/072
Can Ö., Alp E.	Valuation of environmental improvements in a specially protected marine area: A choice experiment approach in Göcek Bay, Turkey	2012	Science of the Total Environment	10.1016/j.scitotenv.2012.09.002
Castaño-Isaza J., Newball R., Roach B., Lau W.W.Y.	Valuing beaches to develop payment for ecosystem services schemes in Colombia's Seaflower marine protected area	2015	Ecosystem Services	10.1016/j.ecoser.2014.10.003
Castellini A., Devenuto L., Ragazzoni A.	The marine environment as tourism-recreational resource. An economic assessment of the demand	2009	New Medit	

Author(s)	Title	Year	Journal title	DOI
Chakrabarty P., Pan S., Mandal R.	Promoting wildlife tourism on geotourism landscape: A study in Manas and Kaziranga National Parks of Assam, India	2019	Geojournal of Tourism and Geosites	10.30892/gtg.24115-352
Chen F., Wu J., Liu J., Hu Y., Chen X., Lim P.-E., Aznan Abdullah W.M., Sjafrie N.D.M., Adirianto B.	Comparison of social-value cognition based on different groups: The case of Pulau Payar in Malaysia and Gili Matra in Indonesia	2019	Ocean and Coastal Management	10.1016/j.ocecoaman.2019.02.010
Chiou C.-R., Lin J.-C., Liu W.-Y., Lin T.-W.	Assessing the recreational value of protective forests at Taitung Forest Park in Taiwan	2016	Tourism Economics	10.5367/te.2015.0468
Chopra K., Adhikari S.K.	Environment development linkages: Modelling a wetland system for ecological and economic value	2004	Environment and Development Economics	10.1017/S1355770X03001037
Christie M., Remoundou K., Siwicka E., Wainwright W.	Valuing marine and coastal ecosystem service benefits: Case study of St Vincent and the Grenadines' proposed marine protected areas	2015	Ecosystem Services	10.1016/j.ecoser.2014.10.002
Conti E., Lexhagen M.	Instagramming nature-based tourism experiences: a netnographic study of online photography and value creation	2020	Tourism Management Perspectives	10.1016/j.tmp.2020.100650
Crespo-Cebada E., Díaz-Caro C., Robina-Ramírez R., Sánchez-Hernández M.I.	Is biodiversity a relevant attribute for assessing natural parks? Evidence from Cornalvo natural park in Spain	2020	Forests	10.3390/F11040410
Cunha J., Elliott M., Ramos S.	Linking modelling and empirical data to assess recreation services provided by coastal habitats: The case of NW Portugal	2018	Ocean and Coastal Management	10.1016/j.ocecoaman.2017.12.022
Czeszczewik D., Ginter A., Mikusiński G., Pawłowska A., Kałuża H., Smithers R.J., Walankiewicz W.	Birdwatching, logging and the local economy in the Białowieża Forest, Poland	2019	Biodiversity and Conservation	10.1007/s10531-019-01808-6
Dagiliūtė R., Žalandauskas M., Sujetovienė G., Žaltauskaitė J.	Willingness to pay for the authenticity of the Curonian spit	2017	Environmental Processes	10.1007/s40710-017-0238-7
Daly C.A.K., Fraser G., Snowball J.D.	Willingness to pay for marine-based tourism in the Ponta do Ouro Partial Marine Reserve, Mozambique	2015	African Journal of Marine Science	10.2989/1814232X.2015.1012556
Dehghani M., Farshchi P., Danekar A., Karami M., Aleshikh A.A.	Recreation value of hara biosphere reserve using willingness-to-pay method	2010	International Journal of Environmental Research	

Author(s)	Title	Year	Journal title	DOI
Dicken M.L.	Socio-economic aspects of boat-based ecotourism during the sardine run within the Pondoland marine protected area, South Africa	2010	African Journal of Marine Science	10.2989/1814232X.2010.502642
Dicken M.L., Hosking S.G.	Socio-economic aspects of the tiger shark diving industry within the Aliwal Shoal marine protected area, South Africa	2009	African Journal of Marine Science	10.2989/AJMS.2009.31.2.10.882
Do Y., Kim J.Y.	An assessment of the aesthetic value of protected wetlands based on a photo content and its metadata	2020	Ecological Engineering	10.1016/j.ecoleng.2020.105816
Dong C.-M., Lin C.-C., Lin S.-P.	Study on the appraisal of tourism demands and recreation benefits for Nanwan Beach, Kenting, Taiwan	2018	Environments - MDPI	10.3390/environments5090097
Dong X., Zhang J., Zhi R., Zhong S., Li M.	Measuring recreational value of world heritage sites based on contingent valuation method: A case study of Jiuzhaigou	2011	Chinese Geographical Science	10.1007/s11769-011-0445-5
Du P., Zhang C., Yang L.	The impact of perceived value and group norm on environmentally friendly behavior of mangrove reserves in coastal cities: The mediating role of tourism satisfaction	2020	Journal of Coastal Research	10.2112/JCR-SI111-043.1
Du Preez M., Dicken M., Hosking S.G.	The value of tiger shark diving within the aliwal shoal marine protected area: A travel cost analysis	2012	South African Journal of Economics	10.1111/j.1813-6982.2011.01292.x
Edge A., Henley M., Daday J., Schulte B.A.	Examining Human Perception of Elephants and Large Trees for Insights Into Conservation of an African Savanna Ecosystem	2017	Human Dimensions of Wildlife	10.1080/10871209.2017.1298168
Esfehani M.H., Albrecht J.N.	Roles of intangible cultural heritage in tourism in natural protected areas	2018	Journal of Heritage Tourism	10.1080/1743873X.2016.1245735
Estifanos T., Polyakov M., Pandit R., Hailu A., Burton M.	What are tourists willing to pay for securing the survival of a flagship species? The case of protection of the Ethiopian wolf	2021	Tourism Economics	10.1177/1354816619880430
Font A.R.	Mass tourism and the demand for protected natural areas: A travel cost approach	2000	Journal of Environmental Economics and Management	10.1006/jeem.1999.1094
Frontuto V., Dalmazzone S., Vallino E., Giaccaria S.	Earmarking conservation: Further inquiry on scope effects in stated preference methods applied to nature-based tourism	2017	Tourism Management	10.1016/j.tourman.2016.11.017

Author(s)	Title	Year	Journal title	DOI
Füzyová L., Lániková D., Novorolský M.	Economic valuation of Tatras National Park and regional environmental policy	2009	Polish Journal of Environmental Studies	
García-Llorente M., Martín-López B., Nunes P.A.L.D., González J.A., Alcorlo P., Montes C.	Analyzing the social factors that influence willingness to pay for invasive alien species management under two different strategies: Eradication and prevention	2011	Environmental Management	10.1007/s00267-011-9646-z
Gelcich S., Amar F., Valdebenito A., Castilla J.C., Fernandez M., Godoy C., Biggs D.	Financing marine protected areas through visitor fees: Insights from tourists willingness to pay in Chile	2013	Ambio	10.1007/s13280-013-0453-z
González R.M., Marrero Á.S., Navarro-Ibáñez M.	Tourists' travel time values using discrete choice models: the recreational value of the Teide National Park	2018	Journal of Sustainable Tourism	10.1080/09669582.2018.1527342
Gürlük S., Rehber E.	A travel cost study to estimate recreational value for a bird refuge at Lake Manyas, Turkey	2008	Journal of Environmental Management	10.1016/j.jenvman.2007.07.017
Hazen H.	Valuing natural heritage: Park visitors' values related to World Heritage sites in the USA	2009	Current Issues in Tourism	10.1080/13683500802538260
Hein L.	Economic benefits generated by protected areas: The case of the Hoge Veluwe Forest, the Netherlands	2011	Ecology and Society	10.5751/ES-04119-160213
Heyes C., Heyes A.	Willingness to pay versus willingness to travel: Assessing the recreational benefits from Dartmoor National Park	1999	Journal of Agricultural Economics	10.1111/j.1477-9552.1999.tb00799.x
Iranah P., Lal P., Wolde B.T., Burli P.	Valuing visitor access to forested areas and exploring willingness to pay for forest conservation and restoration finance: The case of small island developing state of Mauritius	2018	Journal of Environmental Management	10.1016/j.jenvman.2018.07.008
Ison S., Ison T., Marti-Puig P., Needham K., Tanner M.K., Roberts J.M.	Tourist Preferences for Seamount Conservation in the Galapagos Marine Reserve	2021	Frontiers in Marine Science	10.3389/fmars.2020.602767
Jaung W., Carrasco L.R.	Travel cost analysis of an urban protected area and parks in Singapore: a mobile phone data application	2020	Journal of Environmental Management	10.1016/j.jenvman.2020.110238
Juutinen A., Mitani Y., Mäntymaa E., Shoji Y., Siikamäki P., Svento R.	Combining ecological and recreational aspects in national park management: A choice experiment application	2011	Ecological Economics	10.1016/j.ecolecon.2011.02.006

Author(s)	Title	Year	Journal title	DOI
Karahalil U., Başkent E.Z., Köse S.	Integrating visitor characteristics and preferences into forest management plans in protected areas: A case study in Köprülü Canyon National Park	2015	Eco.mont	10.1553/eco.mont-7-2s5
Lal P., Wolde B., Masozera M., Burli P., Alavalapati J., Ranjan A., Montambault J., Banerjee O., Ochuodho T., Mugabo R.	Valuing visitor services and access to protected areas: The case of Nyungwe National Park in Rwanda	2017	Tourism Management	10.1016/j.tourman.2017.01.019
Lara-Pulido J.A., Mojica Á., Bruner A., Guevara-Sanginés A., Simon C., Vásquez-Lavin F., González-Baca C., Infanzón M.J.	A business case for marine protected areas: Economic valuation of the reef attributes of cozumel island	2021	Sustainability (Switzerland)	10.3390/su13084307
Latinopoulos D.	The role of ecotourism in the Prespa National Park in Greece. Evidence from a travel cost method and hoteliers' perceptions	2019	Journal of Environmental Management and Tourism	10.14505/jemt.v10.8(40).03
Lee C.-H., Chen Y.-J., Chen C.-W.	Assessment of the economic value of ecological conservation of the Kenting coral reef	2019	Sustainability (Switzerland)	10.3390/su11205869
León C.J., de León J., Araña J.E., González M.M.	Tourists' preferences for congestion, residents' welfare and the ecosystems in a national park	2015	Ecological Economics	10.1016/j.ecolecon.2015.07.003
Liston-Heyes C., Heyes A.	Recreational benefits from the Dartmoor National Park	1999	Journal of Environmental Management	10.1006/jema.1998.0244
Liu W.-Y., Chen P.-Z., Hsieh C.-M.	Assessing the recreational value of a National Forest Park from ecotourists' perspective in Taiwan	2019	Sustainability (Switzerland)	10.3390/su11154084
Lopes P.F.M., Villasante S.	Paying the price to solve fisheries conflicts in Brazil's Marine Protected Areas	2018	Marine Policy	10.1016/j.marpol.2018.03.016
Louda J., Vojáček O., Slavíková L.	Achieving robust and socially acceptable environmental policy recommendations: Lessons from combining the choice experiment method and institutional analysis focused on cultural ecosystem services	2021	Forests	10.3390/f12040484

Author(s)	Title	Year	Journal title	DOI
Mancini F., Coghill G.M., Lusseau D.	Quantifying wildlife watchers' preferences to investigate the overlap between recreational and conservation value of natural areas	2019	Journal of Applied Ecology	10.1111/1365-2664.13274
Mandziuk A., Parzych S., Studnicki M.	Benefits of recreation in the "nad tanwią" nature reserve determined by the travel cost method	2020	Baltic Forestry	10.46490/BF407
Martín-López B., Gómez-Baggethun E., Lomas P.L., Montes C.	Effects of spatial and temporal scales on cultural services valuation	2009	Journal of Environmental Management	10.1016/j.jenvman.2008.03.013
Martínez Pastur G., Peri P.L., Lencinas M.V., García-Llorente M., Martín-López B.	Spatial patterns of cultural ecosystem services provision in Southern Patagonia	2016	Landscape Ecology	10.1007/s10980-015-0254-9
Marzetti Dall'Aste Brandolini S.	Investing in biodiversity: The recreational value of a natural coastal area	2006	Chemistry and Ecology	10.1080/02757540600720227
Mathieu L.F., Langford I.H., Kenyon W.	Valuing marine parks in a developing country: A case study of the Seychelles	2003	Environment and Development Economics	10.1017/S1355770X03000196
Matthew N.K., Shuib A., Ramachandran S., Mohammad-Afandi S.H.	Economic valuation using travel cost method (TCM) in Kilim Karst Geoforest Park, Langkawi, Malaysia	2019	Journal of Tropical Forest Science	10.26525/jtfs2019.31.1.078089
Mayer M.	Can nature-based tourism benefits compensate for the costs of national parks? A study of the Bavarian Forest National Park, Germany	2014	Journal of Sustainable Tourism	10.1080/09669582.2013.871020
Menkhaus S., Lober D.J.	International ecotourism and the valuation of tropical rainforests in Costa Rica	1996	Journal of Environmental Management	10.1006/jema.1996.0031
Mercer E., Kramer R., Sharma N.	Rain forest tourism - estimating the benefits of tourism development in a new national park in Madagascar	1995	Journal of Forest Economics	
Molina J.R., González-Cabán A., Rodríguez y Silva F.	Wildfires impact on the economic susceptibility of recreation activities: Application in a Mediterranean protected area	2019	Journal of Environmental Management	10.1016/j.jenvman.2019.05.131
Moran D.	Contingent valuation and biodiversity: measuring the user surplus of Kenyan protected areas	1994	Biodiversity and Conservation	10.1007/BF00126859
Mrotek A., Anderson C.B., Valenzuela A.E.J., Manak L., Weber A., Van Aert P., Malizia M., Nielsen E.A.	An evaluation of local, national and international perceptions of benefits and threats to nature in Tierra del Fuego National Park (Patagonia, Argentina)	2019	Environmental Conservation	10.1017/S0376892919000250

Author(s)	Title	Year	Journal title	DOI
Mukanjari S., Muchapondwa E., Demeke E.	Recreation demand and pricing policy for international tourists in developing countries: evidence from South Africa	2020	Journal of Environmental Economics and Policy	10.1080/21606544.2020.1853609
Müller S.M., Peisker J., Bieling C., Linnemann K., Reidl K., Schmieder K.	The importance of cultural ecosystem services and biodiversity for landscape visitors in the biosphere reserve Swabian Alb (Germany)	2019	Sustainability (Switzerland)	10.3390/su11092650
Muñoz L., Hausner V., Brown G., Runge C., Fauchald P.	Identifying spatial overlap in the values of locals, domestic- and international tourists to protected areas	2019	Tourism Management	10.1016/j.tourman.2018.07.015
Mwebaze P., MacLeod A.	Valuing marine parks in a small island developing state: A travel cost analysis in Seychelles	2013	Environment and Development Economics	10.1017/S1355770X12000538
Naidoo R., Adamowicz W.L.	Biodiversity and nature-based tourism at forest reserves in Uganda	2005	Environment and Development Economics	10.1017/S1355770X0400186X
Navrud S., Mungatana E.D.	Environmental valuation in developing countries: The recreational value of wildlife viewing	1994	Ecological Economics	10.1016/0921-8009(94)90024-8
Navrud S., Vondolia G.K.	Using contingent valuation to price ecotourism sites in developing countries	2005	Tourism	
Nikodinoska N., Foxcroft L.C., Rouget M., Paletto A., Notaro S.	Tourists' perceptions and willingness to pay for the control of Opuntia stricta invasion in protected areas: A case study from South Africa	2014	Koedoe	10.4102/koedoe.v56i1.1214
Nikodinoska N., Paletto A., Franzese P.P., Jonasson C.	Valuation of ecosystem services in protected areas: The case of the Abisko National Park (Sweden)	2015	Journal of Environmental Accounting and Management	10.5890/JEAM.2015.11.005
Nitanan K.M., Shuib A., Sridar R., Kunjuraman V., Zaiton S., Syamsul Herman M.A.	The Total Economic Value of Forest Ecosystem Services in the Tropical Forests of Malaysia	2020	International Forestry Review	10.1505/146554820831255551
Ortega-Becerril J.A., Polo I., Belmonte A.	Waterfalls as Geological Value for Geotourism: the Case of Ordesa and Monte Perdido National Park	2019	Geoheritage	10.1007/s12371-019-00366-1
Pandit R., Dhakal M., Polyakov M.	Valuing access to protected areas in Nepal: The case of Chitwan National Park	2015	Tourism Management	10.1016/j.tourman.2014.12.017
Parsons G.R., Thur S.M.	Valuing changes in the quality of coral reef ecosystems: A stated preference study of	2008	Environmental and Resource Economics	10.1007/s10640-007-9171-y

Author(s)	Title	Year	Journal title	DOI
	SCUBA diving in the Bonaire National Marine Park			
Pavlović S., Belij M., Vesić M., Jovanović S.S., Manojlović I.	Improvement of the relationship between environment and tourism: Case study of the National Park Derdap in Serbia	2020	Mitteilungen der Osterreichischen Geographischen Gesellschaft	10.1553/MOEGG161S251
Peh K.S.-H., Thapa I., Basnyat M., Balmford A., Bhattarai G.P., Bradbury R.B., Brown C., Butchart S.H.M., Dhakal M., Gurung H., Hughes F.M.R., Mulligan M., Pandeya B., Stattersfield A.J., Thomas D.H.L., Walpole M., Merriman J.C.	Synergies between biodiversity conservation and ecosystem service provision: Lessons on integrated ecosystem service valuation from a Himalayan protected area, Nepal	2016	Ecosystem Services	10.1016/j.ecoser.2016.05.003
Perez Loyola R., Wang E., Kang N.	Economic valuation of recreational attributes using a choice experiment approach: An application to the Galapagos Islands	2021	Tourism Economics	10.1177/1354816619885236
Pickering C., Walden-Schreiner C., Barros A., Rossi S.D.	Using social media images and text to examine how tourists view and value the highest mountain in Australia	2020	Journal of Outdoor Recreation and Tourism	10.1016/j.jort.2019.100252
Piñeiro-Corbeira C., Barreiro R., Olmedo M., De la Cruz-Modino R.	Recreational snorkeling activities to enhance seascape enjoyment and environmental education in the Islas Atlánticas de Galicia National Park (Spain)	2020	Journal of Environmental Management	10.1016/j.jenvman.2020.111065
Pires N.M., Garla R.C., Carvalho A.R.	The economic role of sharks in a major ecotourism archipelago in the western South Atlantic	2016	Marine Policy	10.1016/j.marpol.2016.06.016
Piriyapada S., Wang E.	Modeling Willingness to Pay for Coastal Tourism Resource Protection in Ko Chang Marine National Park, Thailand	2015	Asia Pacific Journal of Tourism Research	10.1080/10941665.2014.904806
Platania M., Rizzo M.	Willingness to pay for protected areas: A case of Etna Park	2018	Ecological Indicators	10.1016/j.ecolind.2018.04.079
Pongkijvorasin S., Chotiyaputta V.	Climate change and tourism: Impacts and responses. A case study of Khaoyai National Park	2013	Tourism Management Perspectives	10.1016/j.tmp.2012.10.002

Author(s)	Title	Year	Journal title	DOI
Queiroz R.E., Guerreiro J., Ventura M.A.	Demand of the tourists visiting protected areas in small oceanic islands: the Azores case-study (Portugal)	2014	Environment, Development and Sustainability	10.1007/s10668-014-9516-y
Rathnayake R.M.W.	Pricing the enjoyment of 'elephant watching' at the Minneriya National Park in Sri Lanka: An analysis using CVM	2016	Tourism Management Perspectives	10.1016/j.tmp.2016.01.002
Rathnayake R.M.W.	Turtle watching': A strategy for endangered marine turtle conservation through community participation in Sri Lanka	2016	Ocean and Coastal Management	10.1016/j.ocecoaman.2015.10.014
Ribeiro F.P., Ribeiro K.T.	Participative mapping of cultural ecosystem services in Pedra Branca State Park, Brazil	2016	Natureza e Conservacao	10.1016/j.ncon.2016.09.004
Richardson R.B., Loomis J., Weiler S.	Recreation as a spatial good: Distance effects on changes in recreation visitation and benefits	2006	Review of Regional Studies	
Robles-Zavala E., Chang Reynoso A.G.	The recreational value of coral reefs in the Mexican Pacific	2018	Ocean and Coastal Management	10.1016/j.ocecoaman.2018.02.010
Romagosa F., Miró A., Buchaca T., Ventura M.	Residents' versus visitors' knowledge and valuation of aquatic mountain ecosystems in the Catalan Pyrenees	2020	Mountain Research and Development	10.1659/MRD-JOURNAL-D-19-00040.1
Rossi S.D., Barros A., Walden-Schreiner C., Pickering C.	Using social media images to assess ecosystem services in a remote protected area in the Argentinean Andes	2020	Ambio	10.1007/s13280-019-01268-w
Saayman M.	The non-consumptive value of selected marine species at Table Mountain National Park: An exploratory study	2014	South African Journal of Economic and Management Sciences	10.4102/sajems.v17i2.455
Saayman M., Saayman A.	Is the rhino worth saving? A sustainable tourism perspective	2017	Journal of Sustainable Tourism	10.1080/09669582.2016.1197229
Saayman M., Saayman A.	Who is willing to pay to see the Big 7?	2014	Tourism Economics	10.5367/te.2013.0347
Saayman M., Saayman A., Ferreira M.	The socio-economic impact of the Karoo National Park	2009	Koedoe	10.4102/koedoe.v51i1.158
Samdin Z.	Willingness to pay in Taman Negara: A Contingent Valuation Method	2008	International Journal of Economics and Management	

Author(s)	Title	Year	Journal title	DOI
Samdin Z., Aziz Y.A., Radam A., Yacob M.R.	Sustainability of ecotourism resources at taman negara national park: Contingent valuation method	2013	International Journal of Business and Society	
Saminpanya S., Wisarttavisit P., Chumnankid C.	Development of management guidelines from tourist and stakeholder consultations: The case of dongphayayen-khao yai forest complex, Thailand	2009	Managing Leisure	10.1080/13606710902945042
Scheufele G., Bennett J.	Valuing biodiversity protection: Payment for Environmental Services schemes in Lao PDR	2019	Environment and Development Economics	10.1017/S1355770X19000111
Schutgens M.G., Hanson J.H., Baral N., Ale S.B.	Visitors' willingness to pay for snow leopard Panthera uncia conservation in the Annapurna Conservation Area, Nepal	2019	ORYX	10.1017/S0030605317001636
Sekar N., Weiss J.M., Dobson A.P.	Willingness-to-pay and the perfect safari: Valuation and cultural evaluation of safari package attributes in the Serengeti and Tanzanian Northern Circuit	2014	Ecological Economics	10.1016/j.ecolecon.2013.10.012
Sessions C., Wood S.A., Rabotyagov S., Fisher D.M.	Measuring recreational visitation at U.S. National Parks with crowd-sourced photographs	2016	Journal of Environmental Management	10.1016/j.jenvman.2016.09.018
Sinclair M., Ghermandi A., Sheela A.M.	A crowdsourced valuation of recreational ecosystem services using social media data: An application to a tropical wetland in India	2018	Science of the Total Environment	10.1016/j.scitotenv.2018.06.056
Sinclair M., Mayer M., Woltering M., Ghermandi A.	Valuing nature-based recreation using a crowdsourced travel cost method: A comparison to onsite survey data and value transfer	2020	Ecosystem Services	10.1016/j.ecoser.2020.101165
Soares J.O., Soares F.C.	The Recreational Value of Azibo Beaches: A Case Study in the Interior North of Portugal1 [O Valor Recreativo Das Praias De Azibo: Um Estudo De Caso No Interior Norte De Portugal]	2021	Revista Portuguesa de Estudos Regionais	
Soe Zin W., Suzuki A., Peh K.S.-H., Gasparatos A.	Economic value of cultural ecosystem services from recreation in Popa Mountain National Park, Myanmar: A comparison of two rapid valuation techniques	2019	Land	10.3390/LAND8120194
Suresh K., Wilson C., Quayle A., Khanal U., Managi S.	Which national park attributes attract international tourists? A Sri Lankan case study	2021	Tourism Economics	10.1177/13548166211019865

Author(s)	Title	Year	Journal title	DOI
Teh L.S.L., Teh L.C.L., Jolis G.	An economic approach to marine megafauna conservation in the coral triangle: Marine turtles in Sabah, Malaysia	2018	Marine Policy	10.1016/j.marpol.2017.12.004
Thanh An L., Markowski J., Bartos M., Thoai T.Q., Tuan T.H., Rzenca A.	Tourist and Local Resident Preferences for the Northern Yellow-Cheeked Gibbon (Nomascus annamensis) Conservation Program in the Bach Ma National Park, Central Vietnam	2018	Tropical Conservation Science	10.1177/1940082918776564
Thur S.M.	User fees as sustainable financing mechanisms for marine protected areas: An application to the Bonaire National Marine Park	2010	Marine Policy	10.1016/j.marpol.2009.04.008
Tisma S., Boromisa A.-M., Farkas A., Tolic I.	Socio-economic evaluations of nature protected areas: health first effect	2020	European Journal of Geography	10.48088/ejg.s.tis.11.1.108.123
Torquati B., Tempesta T., Vecchiato D., Venanzi S., Paffarini C.	The value of traditional rural landscape and nature protected areas in tourism demand: A study on agritourists' preferences	2017	Landscape Online	10.3097/LO.201753
Trivourea N.M., Karamanlidis A.A., Tounta E., Dendrinos P., Kotomatas S.	People and the Mediterranean monk seal (Monachus monachus): A study of the socioeconomic impacts of the National Marine Park of Alonissos, Northern Sporades, Greece	2011	Aquatic Mammals	10.1578/AM.37.3.2011.305
Trujillo J.C., Carrillo B., Charris C.A., Velilla R.A.	Coral reefs under threat in a Caribbean marine protected area: Assessing divers' willingness to pay toward conservation	2016	Marine Policy	10.1016/j.marpol.2016.03.003
Trujillo J.C., Navas E.J., Vargas D.M.	Valuing coral reef preservation in a Caribbean marine protected area. Economic impact of scuba diving in corals of Rosario and San Bernardo national natural park, Colombia [Valorar la preservación de los arrecifes de coral en un área marina protegida del caribe. Impacto económico del buceo en los corales de Rosario y el parque nacional natural San Bernardo, Colombia]	2017	Cuadernos de Desarrollo Rural	10.11144/Javeriana.cdr14-79.vcrp
Van Beukering P.J.H., Cesar H.S.J., Janssen M.A.	Economic valuation of the Leuser National Park on Sumatra, Indonesia	2003	Ecological Economics	10.1016/S0921-8009(02)00224-0
Van Marwijk R.B.M., Elands B.H.M., Kampen J.K.,	Public Perceptions of the Attractiveness of Restored Nature	2012	Restoration Ecology	10.1111/j.1526-100X.2011.00813.x

Author(s)	Title	Year	Journal title	DOI
Terlouw S., Pitt D.G., Opdam P.				
Veisten K., Dybedal P., Grue B.	Measuring the economic value of nature and national parks: Indirect valuations from travel cost method and tourism satellite accounts	2012	International Journal of Tourism Policy	10.1504/IJTP.2012.052559
Vieira F.A.S., Bragagnolo C., Correia R.A., Malhado A.C.M., Ladle R.J.	A salience index for integrating multiple user perspectives in cultural ecosystem service assessments	2018	Ecosystem Services	10.1016/j.ecoser.2018.07.009
Viirret E., Raatikainen K.J., Fagerholm N., Käyhkö N., Vihervaara P.	Ecosystem services at the Archipelago Sea Biosphere Reserve in Finland: A visitor perspective	2019	Sustainability (Switzerland)	10.3390/su11020421
Viteri Mejía C., Brandt S.	Managing tourism in the Galapagos Islands through price incentives: A choice experiment approach	2015	Ecological Economics	10.1016/j.ecolecon.2015.05.014
Walpole M.J., Goodwin H.J., Ward K.G.R.	Pricing policy for tourism in protected areas: Lessons from Komodo National Park, Indonesia	2001	Conservation Biology	10.1046/j.1523-1739.2001.99231.x
Walyoto S., Peranginangin J.	Economic analysis of environmental and cultural impacts of the development of palm oil plantation	2018	International Journal of Energy Economics and Policy	
Wang P.-W., Jia J.-B.	Tourists' willingness to pay for biodiversity conservation and environment protection, Dalai Lake protected area: Implications for entrance fee and sustainable management	2012	Ocean and Coastal Management	10.1016/j.ocecoaman.2012.03.001
Widawski K., Jary Z., Oleniewicz P., Owczarek P., Markiewicz-Patkowska J., Zaręba A.	Attractiveness of protected areas for geotourism purposes from the perspective of visitors: The example of Babiogórski National Park (Poland)	2018	Open Geosciences	10.1515/geo-2018-0028
Widawski K., Oleśniewicz P., Rozenkiewicz A., Zareba A., Jandová S.	Protected areas: Geotourist attractiveness for weekend tourists based on the example of Gorcza Nski National Park in Poland	2020	Resources	10.3390/RESOURCES9040035
Witt B.	Tourists'willingness to pay increased entrance fees at Mexican protected areas: A multi-site contingent valuation study	2019	Sustainability (Switzerland)	10.3390/su11113041
Woodfield A., Cowie D.	The milford track: valuation estimates of a recreation good	1977	Australian Journal of Agricultural Economics	10.1111/j.1467-8489.1977.tb00197.x

Author(s)	Title	Year	Journal title	DOI
Xu L., Ao C., Mao B., Cheng Y., Sun B., Wang J., Liu B., Ma J.	Which is more Important, Ecological Conservation or Recreational Service? Evidence from a Choice Experiment in Wetland Nature Reserve Management	2020	Wetlands	10.1007/s13157-020-01348-8
Yee J.Y., Loc H.H., Poh Y.L., Vo-Thanh T., Park E.	Socio-geographical evaluation of ecosystem services in an ecotourism destination: PGIS application in Tram Chim National Park, Vietnam	2021	Journal of Environmental Management	10.1016/j.jenvman.2021.112656
Yu B., Cai Y., Jin L., Du B.	Effects on willingness to pay for marine conservation: Evidence from Zhejiang Province, China	2018	Sustainability (Switzerland)	10.3390/su10072298
Zambrano-Monserrate M.A., Silva-Zambrano C.A., Ruano M.A.	The economic value of natural protected areas in Ecuador: A case of Villamil Beach National Recreation Area	2018	Ocean and Coastal Management	10.1016/j.ocecoaman.2018.02.020
Zhang H., Gao Y., Hua Y., Zhang Y., Liu K.	Assessing and mapping recreationists' perceived social values for ecosystem services in the Qinling Mountains, China	2019	Ecosystem Services	10.1016/j.ecoser.2019.101006

Supplementary Material S5

Table S5: Categories of variables used in the review to organize the database

Number of cate-gory	Name of category	Description of category shown as the question to be answered by the coder	Type of extracted data	Examples / codes of category	Source of data	Inductive process-based development of categories
**Meta-information of the article**						
1	**Year of publication**	Which year was the article published?	Numeric	1990; 2021	Data were extracted from the article.	No
**Biogeographical information**						
2	**Name of protected area(s)**	What's the name of the protected area(s) that was/were investigated?	Text	Black Forest; Kilimanjaro National Park; PAs in Scotland - not specified	Data were extracted from the article.	No
3	**Region of protected area(s)**	Which region is/are the projected area(s) located in?		Africa; Asia; Oceania; Europe; Latin America and the Caribbean; North America (Canada and United States)		
4	**Location of protected area(s)**	Which country / countries is/are the projected area(s) located in?		United States; South Africa; Australia		
5	**Geographical location: Latitude and longitude of investigated protected area**	What's the latitude and latitude of the center point of each investigated protected area?	Numeric	Latitude Kilimanjaro National Park -3.04947 Longitude Kilimanjaro National Park 37.35979	Information was extracted from Google Maps.	

6	**Type of ecosystem of protected area**	Which type(s) of ecosystem is/are investigated?<br>NA indicates that the study took place in a / multiple not specified protected area(s) and hence, type(s) of ecosystems could not be reinvestigated.		"Two archipelagos with coral reefs, mangroves, tropical dry forests, and the rocky and sandy coastlines" (Trujillo et al. 2016)		Yes (Data are not mutually exclusive.)
**Valuation information**						
7	**Main objective of article to conduct valuation**	Why was/were the valuation exercise(s) conducted?<br>First consider the broader objective(s) related to valuation. If broader objective(s) is/are not related to valuation, consider specific objective(s).	Text and then translated to nominal.	"This exploratory study aimed to determine firstly the non-consumptive value of five marine species (whales, the Great White shark, penguins, dolphins and seals) and secondly the sociodemographic and behavioural variables that influence willingness to pay to see these species." (Saayman 2014)	Data were extracted from the article.	Yes (Data are not mutually exclusive.)
8	**Specific valuation method**	Which specific valuation method(s) was/were used following Harrison et al. (2017)?	Text and then translated to nominal.	Contingent method; Photo-series analysis; Choice Experiment		

**9**	**Broad valuation method**	Which broad valuation method(s) was/were used following Harrison et al. (2017)?		Stated preference methods; Preference assessment		
**10**	**Classification of valuation technique / method**	How is/are broad method(s) classified?		Socio-cultural classification; Economic classification; Mixed		
**Data gathering and data analysis**						
**11**	**Target social group**	Was/were the (contextual) value(s) of target groups other than tourists (e.g., citizens, residents, policy-makers, and park staff) identified based on a valuation method?	Dummy variable	1 = yes<br>0 = no	Data were extracted from the article.	No
**12**	**Data type in value elicitation process**	Which type(s) of data was/were collected in the value elicitation process?	Numeric	1 = only quantitative<br>2 = only qualitative, e.g., most Likert scales<br>3 = mixed, i.e., quantitative and qualitative		
**13**	**Value metric in value elicitation process**	Was/were monetary, non-monetary or both value metric(s) collected?		1 = only monetary<br>2 = only non-monetary<br>3 = mixed, i.e., monetary and non-monetary		
**14**	**Data type in data analysis**	Which type(s) of data was/were used in data analysis?		1 = only quantitative<br>2 = only qualitative<br>3 = mixed, i.e., quantitative and qualitative		

**15**	**Value metric in data analysis**	Was/were monetary, non-monetary or both value metric(s) used in data analysis?		1 = only monetary<br>2 = only non-monetary<br>3 = mixed, i.e., monetary and non-monetary		
**Value**						
**16**	**Value type**	Which (contextual) value(s) was/were gathered? State all contextual values mentioned by the author.<br>Definition of contextual value type (mainly to distinguish from transcendental value and value indicator) was based on Kenter et al. (2015).	Text	Recreational value; Economic value; Non-use value; Social value; Intrinsic values	Data were extracted from the article. If a contextual value was not stated in the article, missing information was added. See Methods for procedure.	Yes (Data are not mutually exclusive.)
**17**	**Elements provided by nature**	Which natural, e.g., abiotic, biotic, tangible, and intangible, element(s) of nature or element(s) mediated by nature was/were valued as stated in the article?<br>For Travel cost method, protected area was used as the valued natural aspect by default, if not specified by the article.<br>For contingent valuation, please insert question.<br>For choice experiments, please		Protected area; Seascape; Wolf; Water quality		

		insert all natural elements here.	
18	**Non-natural elements**	Which non-natural element(s) was/were valued? For choice experiments, please insert all non-natural elements here.	Information boards; Tourism facilities; Safari; Crowdedness

References

Díaz, S., S. Demissew, J. Carabias, C. Joly, M. Lonsdale, N. Ash, A. Larigauderie, J.R. Adhikari, et al. 2015. The IPBES Conceptual Framework — connecting nature and people. Current Opinion in Environmental Sustainability 14: 1–16. doi:10.1016/j.cosust.2014.11.002.

Harrison, P.A., R. Dunford, D.N. Barton, E. Kelemen, B. Martín-López, L. Norton, M. Termansen, H. Saarikoski, et al. 2017. Selecting methods for ecosystem service assessment: A decision tree approach. Ecosystem Services 29: 481–498. doi:10.1016/j.ecoser.2017.09.016.

Kenter, J.O., L. O'Brien, N. Hockley, N. Ravenscroft, I. Fazey, K.N. Irvine, M.S. Reed, M. Christie, et al. 2015. What are shared and social values of ecosystems? Ecological Economics 111: 86–99. doi:10.1016/j.ecolecon.2015.01.006.

Saayman, M. 2014. The non-consumptive value of selected marine species at Table Mountain National Park: An exploratory study. South African Journal of Economic and Management Sciences 17: 184–193. doi:10.4102/sajems.v17i2.455.

TEEB. 2010. The Economics of Ecosystems and Biodiversity Ecological and Economic Foundations. Edited by Pushpam Kumar. London and Washington: Earthscan.

Trujillo, J.C., B. Carrillo, C.A. Charris, and R.A. Velilla. 2016. Coral reefs under threat in a Caribbean marine protected area: Assessing divers' willingness to pay toward conservation. Marine Policy 68: 146–154. doi:10.1016/j.marpol.2016.03.003.

Supplementary Material S6

Table S6: Four examples of coded articles, see pages below

References

Harrison, P.A., R. Dunford, D.N. Barton, E. Kelemen, B. Martín-López, L. Norton, M. Termansen, H. Saarikoski, et al. 2017. Selecting methods for ecosystem service assessment: A decision tree approach. *Ecosystem Services* 29: 481–498. doi:10.1016/j.ecoser.2017.09.016.

Kenter, J.O., L. O'Brien, N. Hockley, N. Ravenscroft, I. Fazey, K.N. Irvine, M.S. Reed, M. Christie, et al. 2015. What are shared and social values of ecosystems? *Ecological Economics* 111: 86–99. doi:10.1016/j.ecolecon.2015.01.006.

	Name of category	Use of data (Ori-ginal data: OD; Data Ana-lysis: DA; Data base: DB; Re-vised/ added: RA)	Description of category shown as the question to be answered by the coder (OA) / examples of (sub-)categories (DA / DB)	Type of extracted data	Examples and/or codes of category, if applicable	Example article 1	Example article 2	Example article 3	Example article 4
**Meta-information of the article**	**Author(s)**					Pickering C., Walden-Schreiner C., Barros A., Rossi S.D.	Witt B.	Ison S., Ison T., Marti-Puig P., Needham K., Tanner M.K., Roberts J.M.	Mrotek A., Anderson C.B., Valenzuela A.E.J., Manak L., Weber A., Van Aert P., Malizia M., Nielsen E.A.
	**Title**					Using social media images and text to examine how tourists view and value the highest mountain in Australia	Tourists' willingness to pay increased entrance fees at Mexican protected areas: A multi-site contingent valuation study	Tourist Preferences for Seamount Conservation in the Galapagos Marine Reserve	An evaluation of local, national and international perceptions of benefits and threats to nature in Tierra del Fuego National Park (Patagonia, Argentina)

	**Year of publication**		Which year was the article published?	Numeric	2000; 2021	2020	2019	2021	2019
	**Journal**					Journal of Outdoor Recreation and Tourism	Sustainability (Switzerland)	Frontiers in Marine Science	Environmental Conservation
	**DOI**					10.1016/j.jort.2019.100252	10.3390/su11113041	10.3389/fmars.2020.602767	10.1017/S0376892919000250
**Biogeographical data**	**Name of protected area**	OD	What's the name of the protected area?	Text	Black Forest; Kilimanjaro National Park; Protected Areas in Scotland - not specified	Kosciuszko National Park	Calakmul Biosphere Reserve, Cobá Archaeological Zone, Palenque National Park, Sian Ka'an Biosphere Reserve, and Yum Balam Nature Reserve	Galapagos Marine Reserve	Tierra del Fuego National Park
	**Region of protected area**	OD	Which region(s) is/are the projected area(s) located in?	Text	Africa; Asia; Oceania; Europe; Latin America and the Caribbean; North America (Canada and United States)	Oceania	Latin America and the Caribbean	Latin America and the Caribbean	Latin America and the Caribbean
	**Africa**	DA		Dummy variable	1 = yes<br>0 = no				
	**Asia**	DA		Dummy variable	1 = yes<br>0 = no				
	**Oceania**	DA		Dummy variable	1 = yes<br>0 = no	1			

**Europe**	DA		Dummy variable	1 = yes<br>0 = no				
**Latin America and the Caribbean**	DA		Dummy variable	1 = yes<br>0 = no		1	1	1
**North America**	DA		Dummy variable	1 = yes<br>0 = no				
**Location of protected area**	OD	Which country/countries is/are the projected area(s) located in?	Text	United States; South Africa	Australia	Mexico	Ecuador	Argentina
**Number of investigated protected area(s)**	DA	How many protected areas were mentioned? Name of protected area(s) must be stated to identify geographical location.	Numeric	1;2;3	1	5	1	1
**Protected Area 1 Coordinate: Latitude**	DA		Numeric	Latitude Kilimanjaro National Park -3.04947	-36.243.092	18.498.568	-0.626318	-54.841.197
**Protected Area 1 Co-ordinates: Longitude**	DA		Numeric	Longitude Kilimanjaro National Park 37.35979	148.519.786	-89.556.117	-90.350.466	-68.555.331
**Protected Area 2 Coordinate: Latitude**	DA		Numeric	Latitude Kilimanjaro National Park -3.04947		20.490.926		

	**Protected Area 2 Co-ordinates: Longitude**	DA		Numeric	Longitude Kilimanjaro National Park 37.35979		-87.732.520		
	**Protected Area 3 Coordinate: Latitude**	DA		Numeric	Latitude Kilimanjaro National Park -3.04947		17.484.830		
	**Protected Area 3 Co-ordinates: Longitude**	DA		Numeric	Longitude Kilimanjaro National Park 37.35979		-92.049.246		
	**Protected Area 4 Coordinate: Latitude**	DA		Numeric	Latitude Kilimanjaro National Park -3.04947		19.837.569		
	**Protected Area 4 Co-ordinates: Longitude**	DA		Numeric	Longitude Kilimanjaro National Park 37.35979		-87.638.969		
	**Protected Area 5 Coordinate: Latitude**	DA		Numeric	Latitude Kilimanjaro National Park -3.04947		21.270.526		

**Protected Area 5 Co-ordinates: Longitude**	DA		Numeric	Longitude Kilimanjaro National Park 37.35979		-88.443.382		
**Ecosystem of protected area**	OD	What type(s) of ecosystem was/were investigated? State all types of ecosystem named in the article. NA indicates that the study took place in multiple protected areas and and hence, type(s) of ecosystems could not be reinvestigated.	Text		mountain (broader alpine area (> 1800 m))	Caribbean coast, beaches, rainforest, wetlands and mangrove forests	Seamount	Sub-Antarctic Forest Biome Ecologically, this biome hosts the world's southernmost forests, and TFNP's landscape consists of a diverse mosaic of forests (Nothofagus spp.) and peat bogs (Sphagnum spp.) within a highly variable topography of coastline, mountains, valleys, rivers and lakes.
**Terrestrial eco-system(s)**	DA	see DB for terrestrial ecosystem(s) below	Dummy variable	1 = yes<br>0 = no	1	1		1
**Freshwater eco-system(s)**	DA	see DB for freshwater ecosystem(s) below	Dummy variable	1 = yes<br>0 = no		1		1
**Marine eco-system(s)**	DA	see DB for marine ecosystem(s) below	Dummy variable	1 = yes<br>0 = no		1	1	1

	**Terrestrial ecosystem**	DB	Forest; woods; woodlands	Dummy variable	1 = yes<br>0 = no		1		1
	**Terrestrial ecosystem**	DB	Savanna; wood pasture	Dummy variable	1 = yes<br>0 = no				
	**Terrestrial ecosystem**	DB	Low-shrub vegetation; scrubland; shrubland	Dummy variable	1 = yes<br>0 = no				
	**Terrestrial ecosystem**	DB	Mediterranean ecosystem	Dummy variable	1 = yes<br>0 = no				
	**Terrestrial ecosystem**	DB	Grassland; natural meadows; steppe	Dummy variable	1 = yes<br>0 = no				
	**Terrestrial ecosystem**	DB	Heathland	Dummy variable	1 = yes<br>0 = no				
	**Terrestrial ecosystem**	DB	Alpine, subalpine, subnival vegetation	Dummy variable	1 = yes<br>0 = no	1			
	**Terrestrial ecosystem**	DB	Peatland; peat bog; moorland	Dummy variable	1 = yes<br>0 = no				1
	**Terrestrial ecosystem**	DB	Ice fields; glacier	Dummy variable	1 = yes<br>0 = no				
	**Terrestrial ecosystem**	DB	Desert	Dummy variable	1 = yes<br>0 = no				
	**Terrestrial ecosystem**	DB	Tundra	Dummy variable	1 = yes<br>0 = no				
	**Terrestrial ecosystem**	DB	Mountain ecosystem(s)	Dummy variable	1 = yes<br>0 = no	1			1
	**Terrestrial ecosystem**	DB	Archipelago; island	Dummy variable	1 = yes<br>0 = no				
	**Terrestrial ecosystem**	DB	Beach; dunes; sand; coast at the sea	Dummy variable	1 = yes<br>0 = no		1		1
	**Terrestrial ecosystem**	DB	Terrestrial unspecified	Dummy variable	1 = yes<br>0 = no				

	**Terrestrial ecosystem**	DB	Semi-natural ecosystem (e.g., cultural landscape; grazing areas; heathland; agriculturally used ecosystems)	Dummy variable	1 = yes<br>0 = no				
	**Terrestrial ecosystem**	DB	Non-natural ecosystem	Dummy variable	1 = yes<br>0 = no				
	**Terrestrial ecosystem**	DB	Geological rock formations; rocky outcrops; volcano; geysers	Dummy variable	1 = yes<br>0 = no				
	**Freshwater ecosystem**	DB	Lakes; rivers; freshwater water bodies	Dummy variable	1 = yes<br>0 = no				1
	**Freshwater ecosystem**	DB	Estuarine	Dummy variable	1 = yes<br>0 = no				
	**Freshwater ecosystem**	DB	Freshwater wetland	Dummy variable	1 = yes<br>0 = no		1		
	**Freshwater ecosystem**	DB	Freshwater unspecified	Dummy variable	1 = yes<br>0 = no				
	**Freshwater ecosystem**	DB	Marsh	Dummy variable	1 = yes<br>0 = no				
	**Freshwater ecosystem**	DB	Saltwater wetland	Dummy variable	1 = yes<br>0 = no				
	**Marine ecosystem**	DB	Beach; dunes; sand; coast at the sea	Dummy variable	1 = yes<br>0 = no		1		1
	**Marine ecosystem**	DB	Estuarine; coastal wetland; salt marshes; salt water wetland	Dummy variable	1 = yes<br>0 = no				
	**Marine ecosystem**	DB	Mangrove	Dummy variable	1 = yes<br>0 = no		1		

	**Marine ecosystem**	DB	Coral Reef	Dummy variable	1 = yes<br>0 = no				
	**Marine ecosystem**	DB	Seagrass	Dummy variable	1 = yes<br>0 = no				
	**Marine ecosystem**	DB	Marine unspecified	Dummy variable	1 = yes<br>0 = no				
	**Marine ecosystem**	DB	Open marine water bodies; seamount	Dummy variable	1 = yes<br>0 = no			1	
	**Marine ecosystem**	DB	Archipelago; island	Dummy variable	1 = yes<br>0 = no				

**Valuation information**	**Main objective of article to conduct valuation**	OD	Why was/were the valuation exercise(s) conducted?<br>First consider the broader objective(s) related to valuation. If broader objective(s) is/are not related to valuation, consider specific objective(s).	Text		To examine temporal differences in the views and values of tourists associated with locations and activities within a natural area using the content of images and the words used in tags and titles to describe them.	For this reason, a survey of a number of different PA within one country or region, using identical metho-dology, would better identify how visitor WTP varies between sites and what a realistic fee policy might look like. In addition, these stated preference surveys can estimate potential changes in visitation numbers if entrance fees were to be changed. While this type of methodologically consistent, multi-site study is rare, there are a few previous examples from Costa Rica, South Africa, and Tanzania. This study aims to add to this small body of research, with 877 respondents surveyed at five -south-eastern Mexican protected areas, using a double-bounded dichotomous choice contingent valuation survey, combined with extensive questions on visitor characteristics. The use of identical survey methodology at five different sites allows for valid and systematic comparisons between site results.	To date, there is one known valuation study in the Galapagos focusing on ecosystem services provided by mangroves and our research adds further evidence to the policy and conservation-oriented valuations in the management of the Galapagos archipelago (Tanner et al., 2019).<br>(1) Adds to the evidence base on the relative importance of non-use values for Marine Protected Areas.<br>(2) Explores the potential influence of tourists' willingness to pay for seamount conservation in the Galapagos Marine Reserve.<br>(3) Identifies knowledge gaps and recommendations for future research into management measures applicable to seamounts in the Galapagos which we can build on in future research.	In this context, we conducted a socio-cultural evaluation of nature in Tierra del Fuego National Park (TFNP), Argentina. Specifically, a representative sample of Park users was surveyed to determine: (1) their valuation of how the Park contributes to people (i.e., benefits); and (2) their assessment of disturbances that impact the Park (i.e., threats). We compared perceptions between: (1) local<br>Ushuaia residents; (2) national visitors from other Argentine provinces;<br>and (3) international tourists.

	**Value-oriented**	DA	Elicitation / determination of recreational value, economic value, economic benefits, social value, social importance, value to people; comparison of different elements of natures, e.g., ecosystem services; value pluralism, comparing different types of contextual values; value comparison of multiple protected areas	Dummy variable	1 = yes<br>0 = no		1	1	1
	**Tourist-oriented**	DA	Visitor characteristics as predictors; influence of socio-demographic, socio-economic, background information of tourist on valuation of nature; comparison among tourist types; impact of significant attributes, characteristics, quality of protected area on travel decisions and perceptions; impact of entrance fee on travel decisions	Dummy variable	1 = yes<br>0 = no	1			

	**Conser-vation-manage-ment-oriented**	DA	Potential impacts of various pricing strategies on revenues; adaptation of entrance fee; suggestions, guidelines for efficient park management; to address policy implications under alternative park management strategies; developing and maintaining a sustainable tourism; policy development; solution to social conflicts; benefits of management alternatives to improve environmental quality; conservation of nature	Dummy variable	1 = yes<br>0 = no			1	
	**Science-oriented**	DA	Comparison of methods; comparison of data types; combination of data types; new method; valuation tool; methodological and conceptual framework	Dummy variable	1 = yes<br>0 = no				

**Specific valuation method**	OD	Which specific valuation method(s) was/were used following Harrison et al. (2017)?	Text	Contingent method; photo-series analysis; choice Experiment	Flickr photo-series analysis and culturomics analysis of the text	Contingent valuation method (Willingness to pay)	Contingent valuation method (WTP) Ranking	Survey
**Specific method of valuation**	RA		Text		Photo series analysis, Netnography (posts)			Ranking / rating
**Travel cost method**	DA		Dummy variable	1 = yes 0 = no				
**Market price**	DA		Dummy variable	1 = yes 0 = no				
**Choice experiment**	DA		Dummy variable	1 = yes 0 = no				
**Contingent valuation**	DA		Dummy variable	1 = yes 0 = no		1	1	
**Contingent activity method**	DA		Dummy variable	1 = yes 0 = no				
**Ranking / rating**	DA		Dummy variable	1 = yes 0 = no			1	1
**Photo-series analysis**	DA		Dummy variable	1 = yes 0 = no	1			
**Netno-graphy (posts and audio)**	DA		Dummy variable	1 = yes 0 = no	1			
**(Participa-tory) Mapping**	DA		Dummy variable	1 = yes 0 = no				

	**Public Partici-pation Geographic Information Systems**									
	**Interview**	DA		Dummy variable	1 = yes<br>0 = no					
	**Not available**	DA		Dummy variable	1 = yes<br>0 = no					
	**Multiple economic specific methods**	DA	(related to specific valuation method: contingent valuation; choice experiment; travel cost method; market price)	Dummy variable	1 = yes<br>0 = no					
	**Multiple socio-cultural specific methods**	DA	(related to specific valuation method: ranking / rating; interview; (participatory) mapping; Public Participation Geographic Information Systems (PPGIS); contingent activity method; netnography (post and audio); photo-series analysis)	Dummy variable	1 = yes<br>0 = no	1				

**Broad valuation method**	OD	Which broad valuation method(s) was/were used following Harrison et al. (2017)?	Text	Stated preference methods; preference assessment	Photo-series analysis	Stated preference methods	Stated preference methods Preference assessment	Preference assessment
**Broad method of valuation**	RA		Text		Public methods			
**Stated preference methods**	DA	(related to specific valuation methods: contingent valuation, choice experiment)	Dummy variable	1 = yes<br>0 = no		1	1	
**Revealed preference methods**	DA	(related to specific valuation method: travel cost method)	Dummy variable	1 = yes<br>0 = no				
**Market-based method**	DA	(related to specific valuation method: market price)	Dummy variable	1 = yes<br>0 = no				
**Preference assessment**	DA	(related to specific valuation method: ranking / rating)	Dummy variable	1 = yes<br>0 = no			1	1
**Narrative analysis**	DA	(related to specific valuation method: interview)	Dummy variable	1 = yes<br>0 = no				
**(Participa-tory) mapping**	DA	(related to specific valuation methods: (participatory) mapping, Public Participation Geographic Information Systems (PPGIS))	Dummy variable	1 = yes<br>0 = no				

	**Time use method**	DA	(related to specific valuation method: contingent activity method)	Dummy variable	1 = yes<br>0 = no				
	**Public methods**	DA	(related to specific valuation methods: netnography (post and audio); photo-series analysis)	Dummy variable	1 = yes<br>0 = no	1			
	**Not available**			Dummy variable	1 = yes<br>0 = no				
	**Multiple economic broad methods**	DA	(related to broad methods: stated preference methods; revealed preference methods; market-based methods)	Dummy variable	1 = yes<br>0 = no				
	**Multiple socio-cultural methods**	DA	(related to broad valuation methods: (participatory) mapping; narrative analysis; preference assessment; public methods; time use)	Dummy variable	1 = yes<br>0 = no				

	**Classifi-cation of valuation technique / method**	OD	How is/are broad method(s) classified following Harrison et al. (2017)?	Nominal	1 = socio-cultural (beyond strictly monetary measure-ments)<br>2 = economic (monetary measure-ments)<br>3 = mixed, i.e. socio-cultural and economic	1	2	3	1
	**Economic valuation**	DA	(related to broad methods: stated preference methods; revealed preference methods; market-based methods)	Dummy variable	1 = yes<br>0 = no		1		
	**Socio-cultural valuation**	DA	(related to broad valuation methods: (participatory) mapping; narrative analysis; preference assessment; public methods; time use)	Dummy variable	1 = yes<br>0 = no	1			1
	**Mixed economic and socio-cultural methods**	DA		Dummy variable	1 = yes<br>0 = no			1	

**Data gathering and data analysis**	**Target social group**	OD	Was/were the (contextual) value(s) of target groups other than tourists (e.g., citizens, residents, policy-makers, park staff) identified based on a valuation method? If so, state target group(s).	Text	Text				local Ushuaia residents (n = 122)
	**Target social group**	DA		Dummy variable	1 = yes<br>0 = no				1
	**Data type in value elicitation process**	OD / RA / DA	Which type(s) of data was/were collected in the value elicitation process?	Nominal	1 = only quantitative<br>2 = only qualitative<br>3 = mixed, i.e., quantitative and qualitative	2	1	3	2
	**Value metric in value elicitation process**	OD / RA / DA	Was/were monetary, non-monetary, or both value metric(s) collected?	Nominal	1 = only monetary<br>2 = only non-monetary<br>3 = mixed, i.e., monetary and non-monetary	2	1	3	2

	**Data type in Data analysis**	OD / RA / DA	Which type(s) of data was/were used in data analysis?	Nominal	1 = only quantitative<br>2 = only qualitative<br>3 = mixed, i.e., quantitative and qualitative	3	1	1	1
	**Value metric in data analysis**	OD / RA / DA	Was/were monetary, non-monetary, or both value metric(s) used in data analysis?	Nominal	1 = only monetary<br>2 = only non-monetary<br>3 = mixed, i.e., monetary and non-monetary	2	1	3	2

Value									
	**Value type**	OD	Which (contextual) value type(s) was/were investigated? State all contextual value type(s) mentioned by the author(s). Definition of contextual value type was based on Kenter et al. (2015), mainly to distinguish from transcendental value and value indicator.	Text	Recreational value; Economic value; Non-use value; Social value; Intrinsic value	sociocultural value; aesthetic value	WTP value	social value in economic terms: non-use value; Although the use-values (i.e., economic value) of seamount ecosystem services are important components to take into consideration for management, this study considers primarily the non-use values (i.e., social value) provided by Galapagos seamounts using a stated preference survey to understand visitors preferences for conservation of seamounts in the Galapagos Single choice: Reason for expressing a positive willingness to pay: I want to preserve the seamount biodiversity for future generations (bequest value); Because the biodiversity on seamounts is unique (existence value); Because the biodiversity on seamounts has a positive impact on the marine economy (direct-use value); I want to personally contribute to projects that protect the environment (warm glow); Because I can afford it (warm glow); Other (specify) 2	national visitors: highest valued observation, biodiversity, existence; lowest valued: economic, human needs, recreation international visitors: highest valued observation, biodiversity, existence; lowest valued: border protection, human needs, economic Argentine visitors valued the relational benefits of TFNP significantly more than international tourists national visitors valued the Park significantly more for its contribution to local identity and border protection. Argentines reported significantly higher perceived values as well for both history and culture and education and science than international tourists.
	**Value type**	RA		Text			Economic value		
	**Recreation-al value**	DA	Leisure, recreation(al), sightseeing value; observation	Dummy variable	1 = yes<br>0 = no				1
	**Leisure value**	DB		Dummy variable	1 = yes<br>0 = no				

Recreation-(al) value	DB		Dummy variable	1 = yes<br>0 = no				1
Sightseeing value								
Observation	DB		Dummy variable	1 = yes<br>0 = no				1
**Tourism value**	DA	Tourism, tourist(ic) value	Dummy variable	1 = yes<br>0 = no				
**Conserva-tion value**	DA	Conservation value	Dummy variable	1 = yes<br>0 = no				
**Economic value**	DA	Welfare, gross benefit, consumer surplus, economic, monetary, marketing value	Dummy variable	1 = yes<br>0 = no		1	1	1
Welfare value	DB		Dummy variable	1 = yes<br>0 = no				
Gross benefit value	DB		Dummy variable	1 = yes<br>0 = no				
Consumer surplus value	DB		Dummy variable	1 = yes<br>0 = no				
Economic / monetary value	DB		Dummy variable	1 = yes<br>0 = no		1	1	1
Marketing value	DB		Dummy variable	1 = yes<br>0 = no				
**Direct use value**	DA	Direct use, non-consumptive (use), recreational(al) use value; use value from recreation	Dummy variable	1 = yes<br>0 = no			1	
**Indirect use value**	DA	Indirect use value	Dummy variable	1 = yes<br>0 = no				

	**Bequest (non-use) value**	DA	Bequest non-use value	Dummy variable	1 = yes<br>0 = no			1	
	**Existence (non-use) value**	DA	Existence non-use value	Dummy variable	1 = yes<br>0 = no			1	
	**Option (use) value**	DA		Dummy variable	1 = yes<br>0 = no				
	**Instrumen-tal value**	DA	Subsistence, instrumental value; human needs	Dummy variable	1 = yes<br>0 = no				1
	**Subsistenc e**	DB		Dummy variable	1 = yes<br>0 = no				
	**Instrumenta l value**	DB		Dummy variable	1 = yes<br>0 = no				1
	**Human needs**	DB		Dummy variable	1 = yes<br>0 = no				1
	**Socio-cultural value**	DA	Experience, perceived, extrinsic, socio-cultural value	Dummy variable	1 = yes<br>0 = no	1			
	**Socio-cultural value**	DB		Dummy variable	1 = yes<br>0 = no	1			
	**Extrinsic value**	DB		Dummy variable	1 = yes<br>0 = no				
	**Experience value**	DB		Dummy variable	1 = yes<br>0 = no				
	**Perceived value**	DB		Dummy variable	1 = yes<br>0 = no				
	**Cultural value**	DA	Cultural diversity, cultural value	Dummy variable	1 = yes<br>0 = no				1
	**Cultural diversity**	DB		Dummy variable	1 = yes<br>0 = no				1

	Cultural value	DB		Dummy variable	1 = yes<br>0 = no				
	**Social value**	DA	Social value	Numeric	>0 = yes<br>0 = no			1	
	**Aesthetic value**	DA	Aesthetics, aesthetical, attractiveness, landscape value	Dummy variable	1 = yes<br>0 = no	1			
	Aesthetics value	DB	Aesthetics, aesthetical value	Dummy variable	1 = yes<br>0 = no	1			
	Attractive-ness value	DB		Dummy variable	1 = yes<br>0 = no				
	Landscape value	DB		Dummy variable	1 = yes<br>0 = no				
	**Local identity**	DA	Local identity	Dummy variable	1 = yes<br>0 = no				1
	**Other contextual value**	DA	Future, eudemonic, utopian, epistemic, emotional value; border protection	Dummy variable	1 = yes<br>0 = no				1
	Future value	DB		Dummy variable	1 = yes<br>0 = no				
	Eudemonic value	DB		Dummy variable	1 = yes<br>0 = no				
	Utopian value	DB		Dummy variable	1 = yes<br>0 = no				
	Epistemic value	DB		Dummy variable	1 = yes<br>0 = no				
	Emotional value	DB		Dummy variable	1 = yes<br>0 = no				
	Border protection	DB		Dummy variable	1 = yes<br>0 = no				1
	**Spiritual value**	DA	Religious, spiritual value	Dummy variable	1 = yes<br>0 = no				1

	**Spiritual value**	DB		Dummy variable	1 = yes<br>0 = no				
	**Religious value**	DB		Dummy variable	1 = yes<br>0 = no				
	**Learning value**	DA	Cognitive, scientific, educational, learning, inspiration value	Dummy variable	1 = yes<br>0 = no				1
	**Cognitive value**	DB		Dummy variable	1 = yes<br>0 = no				
	**Scientific value**	DB		Dummy variable	1 = yes<br>0 = no				1
	**Educational value**	DB		Dummy variable	1 = yes<br>0 = no				
	**Learning value**	DB		Dummy variable	1 = yes<br>0 = no				1
	**Inspiration value**	DB		Dummy variable	1 = yes<br>0 = no				
	**Therapeutic value**	DA	Therapeutic value	Dummy variable	1 = yes<br>0 = no				1
	**Historic value**	DA	Historic value	Dummy variable	1 = yes<br>0 = no				1
	**Relational value**	DA	Relational value	Dummy variable	1 = yes<br>0 = no				1
	**Iconic value**	DA	Outstanding universal, importance, authenticity value	Dummy variable	1 = yes<br>0 = no				
	**Outstanding universal value**	DB		Dummy variable	1 = yes<br>0 = no				
	**Importance value**								
	**Authenticity value**	DB		Dummy variable	1 = yes<br>0 = no				

	**Existence value**	DA	Existence value	Dummy variable	1 = yes<br>0 = no				1
	**Intrinsic value**	DA	Ecological, biocentric, life sustaining, biological diversity, biodiversity, environmental, intrinsic value	Dummy variable	1 = yes<br>0 = no				1
	**Ecological value**	DB		Dummy variable	1 = yes<br>0 = no				
	**Biocentric value**	DB		Dummy variable	1 = yes<br>0 = no				
	**Life sustaining value (intrinsic)**	DB		Dummy variable	1 = yes<br>0 = no				
	**Natural value**	DB							
	**Biological diversity / Biodiversity value (Intrinsic)**	DB		Dummy variable	1 = yes<br>0 = no				1
	**Environ-mental value**	DB		Dummy variable	1 = yes<br>0 = no				
	**Intrinsic value**	DB		Dummy variable	1 = yes<br>0 = no				1

**Object of value: nature**	OD	Which natural, e.g., abiotic, biotic, tangible, and intangible, element(s) of nature or element(s) mediated by nature was/were valued as stated in the article?<br>For Travel cost method, protected area was used as the valued natural element by default, if not specified by the article.<br>For contingent valuation, please insert question.<br>For choice experiments, please insert all natural elements here.	Text	Protected area; Seascape; Wolf; Water quality	landscapes, geological features, fauna and flora	protected area entrance fees were first asked if they would be willing to pay a specified price p to enter the site;	Protected area (deep-sea ecosystems, species, biodiversity)<br>The Galapagos National Park is under the management of the Galapagos National Park Directorate. In the near future, they are proposing to increase the tourist entry fee in order to allocate more funds for deep-sea seamount conservation. The generated funds will be used for seamounts conservation projects, education and outreach [. . .] Please select the maximum amount you would definitely be willing to pay (USD) in additional entrance fees to fund seamount biodiversity conservation programs, education and outreach.	material, non-material and regulating benefits providing potable water; other human needs, such as food, firewood, etc.; economic resources; regulation of natural phenomena, such as hydrological cycles, erosion observing nature; recreation and picnicking; historical and cultural values; education and science; local identity; spiritual values; border protection biodiversity; existence
**Biocultural elements**	DA	Heritage; folklore; traditions; art	Dummy variable	1 = yes<br>0 = no				
**Protected area**	DA	Protected area (per se and/or increase in its size)	Dummy variable	1 = yes<br>0 = no		1	1	
**Land-/ seascape**	DA	Landscape; seascape; scenery	Dummy variable	1 = yes<br>0 = no	1		1	

	**Landscape**	DB	Landscape	Dummy variable	1 = yes<br>0 = no	1			
	**Scenery**	DB	Scenery	Dummy variable	1 = yes<br>0 = no				
	**Seascape**	DB	Seascape	Dummy variable	1 = yes<br>0 = no			1	
	**Sound-scape**	DA	Soundscape	Dummy variable	1 = yes<br>0 = no				
	**Smellscape**	DA	Smell	Dummy variable	1 = yes<br>0 = no				
	**Non-natural ecosystem**	DA	Non-natural ecosystem	Dummy variable	1 = yes<br>0 = no				
	**Semi-natural ecosystem**	DA	Semi-natural ecosystem	Dummy variable	1 = yes<br>0 = no				
	**Ecosystem**	DA	Forest; grassland; wetland; pasture; meadows; freshwater bodies; desert; steppe; tundra; open woodland; savanna; Mediterranean ecosystem (fynbos; nama-karoo; succulent-karoo; thickets); mountain; estuaries; glacier; beach; coral reef; marine forest; mangrove; island; archipelago; coast	Dummy variable	1 = yes<br>0 = no				
	**Forest**	DB	Forest	Dummy variable	1 = yes<br>0 = no				
	**Grassland**	DB	Grassland	Dummy variable	1 = yes<br>0 = no				

	**Wetland**	DB	Wetland	Dummy variable	1 = yes<br>0 = no				
	**Pasture, meadows**	DB	Pasture; meadows	Dummy variable	1 = yes<br>0 = no				
	**Freshwater bodies**	DB	Freshwater bodies	Dummy variable	1 = yes<br>0 = no				
	**Desert steppe**	DB	Desert steppe	Dummy variable	1 = yes<br>0 = no				
	**Tundra**	DB	Tundra	Dummy variable	1 = yes<br>0 = no				
	**Open woodland**	DB	Open woodland	Dummy variable	1 = yes<br>0 = no				
	**Savanna**	DB	Savanna	Dummy variable	1 = yes<br>0 = no				
	**Mediterra-nean eco-system(s)**	DB	Mediterranean ecosystem (Fynbos; Nama-karoo; Succulent-karoo; Thickets)	Dummy variable	1 = yes<br>0 = no				
	**Mountain**	DB	Mountain	Dummy variable	1 = yes<br>0 = no				
	**Estuaries**	DB	Estuaries	Dummy variable	1 = yes<br>0 = no				
	**Glacier**	DA	Glacier	Dummy variable	1 = yes<br>0 = no				
	**Beach**	DB	Beach	Dummy variable	1 = yes<br>0 = no				
	**Coral reef**	DB	Coral reef	Dummy variable	1 = yes<br>0 = no				
	**Marine forest**	DB	Marine forest	Dummy variable	1 = yes<br>0 = no				

	**Mangrove**	DB	Mangrove	Dummy variable	1 = yes<br>0 = no				
	**Island, archipelago**	DB	Island; archipelago	Dummy variable	1 = yes<br>0 = no				
	**Coast**	DB	Coast	Dummy variable	1 = yes<br>0 = no				
	**Ecological quality and functioning**	DA	Ecological health, integrity, quality; visibility; Supporting Ecosystem Service(s)	Dummy variable	1 = yes<br>0 = no				
	**Cultural Ecosystem Service(s) / non-material Nature's Contribu-tions to**	DA	Cultural Ecosystem Service(s); non-material Nature's Contribu-tions to People	Dummy variable	1 = yes<br>0 = no				1
	**Regulating Service(s) / regulating Nature's Contribu-tions to People**	DA	Regulating Service(s); regulating Nature's Contributions to People	Dummy variable	1 = yes<br>0 = no				1
	**Provisio-ning Service(s) / material Nature's Contribu-tions to People**	DA	Provisioning Service(s); material Nature's Contributions to People	Dummy variable	1 = yes<br>0 = no				1

	**Ecosystem Service(s) / Nature's Contribu-tions to People unspecified**	DA	Ecosystem Service(s) / Nature's Contribu-tions to People unspecified	Dummy variable	1 = yes<br>0 = no				
	**Animal and plant diversity**	DA	Flora and fauna: Biodiversity; species richness	Dummy variable	1 = yes<br>0 = no	1			1
	**Endangered animal and plant species**	DA	Endangered animal and plant species	Dummy variable	1 = yes<br>0 = no				
	**Wildlife**	DA	Wildlife	Dummy variable	1 = yes<br>0 = no				
	**Invasive species**	DA	Invasive species	Dummy variable	1 = yes<br>0 = no				1
	**Protected species**	DA	Protected; rare; endangered species	Dummy variable	1 = yes<br>0 = no				
	**Animal diversity**	DA	Biodiversity; species composition; functional group; several species; species richness (number of species); unspecified	Dummy variable	1 = yes<br>0 = no				
	**Bio-diversity, species composition**	DB	Biodiversity; species composition	Dummy variable	1 = yes<br>0 = no				
	**Functional group**	DB	Functional group	Dummy variable	1 = yes<br>0 = no				

**Several species**	DB	Several species	Dummy variable	1 = yes<br>0 = no				
**Species richness (Number of species)**	DB	Species richness (Number of species)	Dummy variable	1 = yes<br>0 = no				
**Unspecified**	DB	Unspecified	Dummy variable	1 = yes<br>0 = no				
**Mammal**	DA	Mammal	Dummy variable	1 = yes<br>0 = no				1
**Reptile**	DA	Reptile	Dummy variable	1 = yes<br>0 = no				
**Amphibian**	DA	Amphibian	Dummy variable	1 = yes<br>0 = no				
**Fish**	DA	Fish	Dummy variable	1 = yes<br>0 = no				
**Bird**	DA	Bird	Dummy variable	1 = yes<br>0 = no				
**Vertebrates**	DA	Vertebrates	Dummy variable	1 = yes<br>0 = no			1	
**Invertebra-tes**	DA	Invertebrates	Dummy variable	1 = yes<br>0 = no			1	
**Iconic animal species**	DA	Iconic; flagship species	Dummy variable	1 = yes<br>0 = no				
**Plant diversity**	DA	Unspecified; functional community; several species; biodiversity; species composition	Dummy variable	1 = yes<br>0 = no				
**Unspecified**	DB	Unspecified	Dummy variable	1 = yes<br>0 = no				
**Flora: Functional community**	DB	Flora: Functional community	Dummy variable	1 = yes<br>0 = no				

	**Several species**	DB	Several species	Dummy variable	1 = yes<br>0 = no				
	**Bio-diversity, species composition**	DB	Biodiversity; species composition	Dummy variable	1 = yes<br>0 = no				
	**Single plant species**	DA	Single plant species	Dummy variable	1 = yes<br>0 = no				
	**Geological elements**	DA	Geosites; geological formation and features; waterfalls; geysers and volcanoes	Dummy variable	1 = yes<br>0 = no	1			
	**Abiotic resources**	DA	Minerals, thermal water	Dummy variable	1 = yes<br>0 = no				
	**Weather / climate**	DA	Weather (phenomena); climate; climatic change	Dummy variable	1 = yes<br>0 = no				1
	**Object of value: non-natural elements**	OD	Which non-natural element(s) was/were valued?<br>For choice experiments, please insert all non-natural elements here.	Text	Information boards; Tourism facilities; Safari; Crowdedness	Cultural history, tourism facilities		Please select the maximum amount you would definitely be willing to pay (USD) in additional entrance fees to fund seamount biodiversity conservation programs, education and outreach.	Use by residents<br>Trash, dirt road<br>Tourists
	**Object of value: non-natural elements**	DA		Dummy variable	1 = yes<br>0 = no	1		1	1

	**Learning and recreation programs**	DA	Information boards; walking safari; tours; programs offering nature-based recreational activities; implementation of an environmental education program; education; scientific project	Dummy variable	1 = yes<br>0 = no			1	
	**Mainten-ance of protected area**	DA	Fence the park; employment of more personnel; elimination of wildlife damages, waste	Dummy variable	1 = yes<br>0 = no				1
	**Touristic infrastruc-ture and services**	DA	Infrastructure; facilities; touristic services; benches; size and number of resting places; viewpoints; (hiking) parking places; railway; transportation; hotel; camping; garbage removal; outreach	Dummy variable	1 = yes<br>0 = no	1		1	
	**Cultural landscape and history**	DA	Urban, cultural landscape; artificial scenes; cultural history; cultural heritage; buildings (e.g., farms; garden sheds); traditional architecture	Dummy variable	1 = yes<br>0 = no	1			

	**Economic contribution**	DA	Economic contribution; local industry; business profit; employment; income of individuals; community welfare	Dummy variable	1 = yes<br>0 = no				
	**Traditional knowledge**	DA	Local, traditional knowledge; customs	Dummy variable	1 = yes<br>0 = no				
	**Local interaction**	DA	Local interaction; use by residents	Dummy variable	1 = yes<br>0 = no				
	**Visitors**	DA	Number of visitors; visitor congestion; crowdedness; use by residents, visitor movement	Dummy variable	1 = yes<br>0 = no				1
	**Trip expendi-tures**	DA	Trip expenditures; travel time	Dummy variable	1 = yes<br>0 = no				

Supplementary Material S7

Additional results

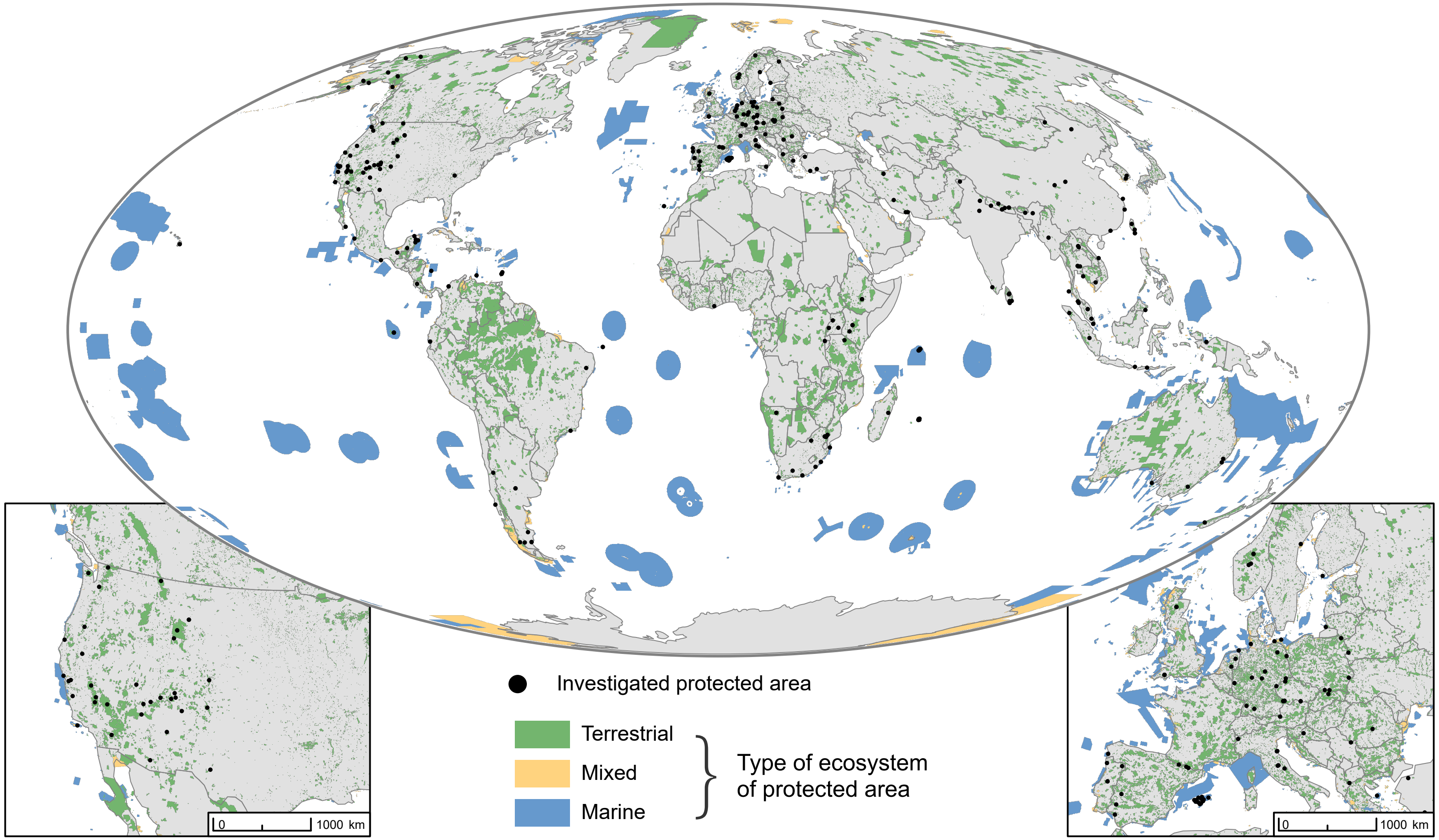

Figure S1: Geographical overview of the protected areas investigated by the articles displayed on the global distribution of publicly available protected areas. The figure was created with the software ARCGis 10.6.1 (Environmental Systems Research Institute ESRI 2018, https://www.esri.com/en-us/home); information on the global distribution of protected areas was sourced from the World Database on Protected Areas (WDPA) (IUCN and UNEP-WCMC 2021) but was not publicly exhaustively available for, e.g., China, India, and Turkey

Table S7: Summary of tangible and intangible elements of nature identified in literature review and number of articles that investigated each element of nature as object of value. Data are not mutually exclusive

Natural element as object of value	Number of articles that investigated natural element
Protected area	100
Cultural ecosystem service(s) / non-material nature's contributions to people	61
Ecosystem	30
Land-/seascape	29
Ecological quality and functioning	25
Animal and plant diversity	26
Iconic animal species	20
Mammal	18
Animal diversity	17
Fish	17
Plant diversity	16
Biocultural elements	14
Geological elements	14
Regulating service(s) / regulating nature's contributions to people	10
Wildlife	10
Provisioning service(s) / material nature's contributions to people	9
Bird	9
Soundscape	5
Invasive animal species	5
Invasive plant species	5
Invertebrates	4
Protected animal species	4
Weather / climate	4
Smellscape	3

Natural element as object of value	Number of articles that investigated natural element
Semi-natural ecosystem	3
Ecosystem Service unspecified	3
Reptile	2
Protected plant species	2
Abiotic elements	2
Non-natural ecosystem	1
Endangered animal and plant species	1
Amphibian	1
Vertebrates	1
Single plant species	1

Table S8: Summary of non-natural elements identified in literature review and number of articles that investigated each non-natural element as object of value. Data are not mutually exclusive

Non-natural element as object of value	Number of articles that investigated non-natural element
Touristic infrastructure and services	24
Learning and recreation programs	14
Cultural landscape and history	12
Use by visitors	11
Traditional knowledge	5
Economic contribution	3
Trip expenditures	3
Maintenance of protected areas	2
Local interaction	2

Table S9: Summary of specific valuation methods identified in literature review and number of articles that applied specific valuation methods. Data are not mutually exclusive

Specific valuation method	Classification of value elicitation	Number of articles that applied specific valuation method
Contingent valuation	Economic	57
Travel cost method	Economic	41
Ranking / rating	Socio-cultural	27
Choice experiment	Economic	17
Photo-series analysis	Socio-cultural	12
Market price	Economic	8
Public Participation Geographic Information Systems (PPGIS)	Socio-cultural	3
Not specified	Socio-cultural	3
Netnographie: social media / online content (posts and audio)	Socio-cultural	3
Interview	Socio-cultural	3
(Participatory) Mapping	Socio-cultural	2
Contingent activity method	Socio-cultural	1

Table S10: Association between value types as identified in the literature review on tourist's valuation of nature in protected areas and the value types embedded in the three most commonly used valuation frameworks: the Total Economic Value (TEV), The Economics of Ecosystems and Biodiversity (TEEB), and the Intergovernmental Science-Policy Platform on Biodiversity and Ecosystem Services (IPBES) valuation framework. This table deepens the information presented in Figure 7 in the main text and shows the quotes found in the literature of this systematic review that prove the association between value types identified in the reviewed literature and those embedded in the three valuation frameworks. The background color represents the degree of association between each value type identified in the literature review and the different value types used in the three valuation frameworks: high association (black background), medium association (dark gray background), low association (light gray background), and no association (white background). Example quotes from articles of systematic literature review are presented in black and white (Black and white were chosen to improve readability against the background.). Examples in green are added by authors of the literature review (Different shades of green were chosen to improve readability against the background.). Biophysical values according to TEEB were not considered, as they cannot be elicited for tourists. We considered that use and non-use values (TEV) measured through non-monetary metrics can represent socio-cultural values (TEEB) in certain cases. For example, the direct use value can be elicited in non-monetary terms by the (additional) time a tourist is willing to travel to reach a protected area, that is, medium association

**Valuation framework** / **Value type**	**TEV**		**TEEB**		**IPBES**		
	Use	Non-use	Economic	Socio-cultural	Intrinsic	Relational	Instrumental
Economic	Beach erosion is an imminent problem in San Andres Island. If the beaches were reduced in size (by half the width), would you come back for another visit? If you would come back, would you be willing to pay the same price you paid this time even if the beaches were smaller by half?	Willingness to pay of tourists to the conservation of whale sharks. (Anna and Saputra 2017)	Willingness to pay of tourists to the conservation of whale sharks. (Anna and Saputra 2017)<br><br>Beach erosion is an imminent problem in San Andres Island. If the beaches were reduced in size (by half the width), would you come back for another visit? If you would come back, would you be willing to pay the same price you paid this time even if the beaches were smaller by half? If you won't pay the	I value it because it provides opportunities for fisheries, minerals and tourism (such as outfitting and guiding). (Chen et al. 2019)			I value it because it provides opportunities for fisheries, minerals and tourism (such as outfitting and guiding). (Chen et al. 2019)

Valuation framework / Value type	TEV		TEEB		IPBES		
	Use	Non-use	Economic	Socio-cultural	Intrinsic	Relational	Instrumental
	If you won't pay the same price for half the beach, how much (%) of your package price would you be willing to pay for your next visit? (Castaño-Isaza et al. 2014)		same price for half the beach, how much (%) of your package price would you be willing to pay for your next visit? (Castaño-Isaza et al. 2014)				
Recre-ational	Travel cost model. (Pongkijvorasin and Chotiyaputta 2013)<br><br>Crowdsourced travel cost model. (Sinclair et al. 2020)<br><br>Willingness to pay for use of the recreation value of forests and natural ecosystems. (Dehghani et al. 2010)		Travel cost model. (Pongkijvorasin and Chotiyaputta 2013)<br><br>Crowdsourced travel cost model. (Sinclair et al. 2020)<br><br>Willingness to pay for use of the recreation value of forests and natural ecosystems. (Dehghani et al. 2010)	Mapping of recreational value: It provides a place for my favorite outdoor recreation activities. (Bogdan et al. 2019)		Expression of the (relational) value on recreation and picnicking based on Likert scale. (Mrotek et al. 2019)	Travel cost model. (Pongkijvorasin and Chotiyaputta 2013)<br><br>Crowdsourced travel cost model. (Sinclair et al. 2020)<br><br>Mapping of recreational value: It provides a place for my favorite outdoor recreation activities. (Bogdan et al. 2019)<br><br>Willingness to pay for use of the recreation value of forests and natural ecosystems. (Dehghani et al. 2010)
Direct use	Willingness to pay for the use of the recreation		Willingness to pay for the preservation or use of the recreation value	How much additional time are you willing to travel to reach Dartmoor			Willingness to pay for the use of the recreation value of forests and

Valuation framework / Value type	TEV		TEEB		IPBES		
	Use	Non-use	Economic	Socio-cultural	Intrinsic	Relational	Instrumental
	value of forests and natural ecosystems. (Dehghani et al. 2010)<br><br>Reason for expressing a positive willingness to pay: Because the biodiversity on seamounts has a positive impact on the marine economy. (Ison et al. 2021)		of forests and natural ecosystems. (Dehghani et al. 2010)<br><br>Reason for expressing a positive willingness to pay: Because the biodiversity on seamounts has a positive impact on the marine economy. (Ison et al. 2021)	National Park? (Heyes and Heyes 1999)			natural ecosystems. (Dehghani et al. 2010)<br><br>Reason for expressing a positive willingness to pay: Because the biodiversity on seamounts has a positive impact on the marine economy. (Ison et al. 2021)
Aesthetic	The aesthetic value of the scenic sights, smells, sounds of the protected area measured as willingness to pay.		The aesthetic value of the scenic sights, smells, sounds of the protected area measured as willingness to pay.	Aesthetic value of protected wetlands based on a photo content and its metadata. (Do and Kim 2020)<br><br>Mapping of aesthetic value: I enjoy the scenery, sights, sounds, smells, etc. (Bogdan et al. 2019)		Aesthetic value of protected wetlands based on a photo content and its metadata (people in the photo). (Do and Kim 2020)<br><br>Mapping of aesthetic value: I enjoy the scenery, sights, sounds, smells, etc. (Bogdan et al. 2019)	Aesthetic value of protected wetlands based on a photo content and its metadata (hints of recreational use). (Do and Kim 2020)
Existence		Willingness to pay of tourists to the conservation of whale sharks.	Willingness to pay of tourists to the conservation of whale sharks. (Anna and Saputra 2017)	Interpretation of Panoramio photographs regarding existence values, which included photos directly related to individual species of	The moral responsibility expressed by a tourist to conserve a particular species because of its right to exist.	The moral responsibility expressed by a tourist to conserve a particular species that derived from the connection with the species.	Willingness to pay of tourists to the conservation of whale sharks. (Anna and Saputra 2017)

Valuation framework / Value type	TEV		TEEB		IPBES		
	Use	Non-use	Economic	Socio-cultural	Intrinsic	Relational	Instrumental
		(Anna and Saputra 2017)		flora and fauna, both native and allochthonous. (Martínez Pastur et al. 2016)			
Intrinsic		Intrinsic value of a species in the protected area expressed by a tourist measured as willingness to conserve the species for its right to exit.	Intrinsic value of a species in the protected area expressed by a tourist measured as willingness to conserve the species for its right to exit.	Expression of the intrinsic value of biodiversity based on Likert scale. (Mrotek et al. 2019)	Expression of the intrinsic value of biodiversity based on Likert scale. (Mrotek et al. 2019)		
Socio-cultural				The respondents were asked to evaluate the importance of each habitat as a producer of different ecosystem services using Likert scale. (Viirret et al. 2019)	The value of biodiversity diversity as expressed by a tourist in terms of its right to exist.	Expression of (relational) values on, e.g., observing nature; recreation and picnicking; historical and cultural values; education and science; local identity; spiritual values, based on Likert scale. (Mrotek et al. 2019)	Expression of (instrumental) values on, e.g., providing potable water; food, firewood, or other economic resources, based on Likert scale. (Mrotek et al. 2019)
Cultural	The cultural value of the scenic sights, smells, sounds of the protected area measured as willingness to pay.	The value of biocultural diversity, measured as willingness to pay to conserve it for future generations.	The cultural value of the scenic sights, smells, sounds of the protected area measured as willingness to pay.	I value it because it is a place for me to continue and pass down the wisdom, knowledge, traditions and way of life of my ancestors. (Chen et al. 2019)<br><br>The respondents were asked to evaluate the	The value of biocultural diversity as expressed by a tourist in terms of its right to exist.	I value it because it is a place for me to continue and pass down the wisdom, knowledge, traditions and way of life of my ancestors. (Chen et al. 2019)	The respondents were asked to evaluate the importance of each studied habitat as a producer of each ES including the significance of the habitat as contributing to local heritage, such as traditional land uses and

Valuation framework / Value type	TEV		TEEB		IPBES		
	Use	Non-use	Economic	Socio-cultural	Intrinsic	Relational	Instrumental
				importance of each studied habitat as a producer of each ES including the significance of the habitat as contributing to local heritage, such as traditional land uses and other cultural practices typical for the Archipelago Sea region. (Viirret et al. 2019)			other cultural practices typical for the Archipelago Sea region. (Viirret et al. 2019)
Bequest (Non-use)		Reason for expressing a positive willingness to pay: I want to preserve the seamount biodiversity for future generations. (Ison et al. 2021)	Reason for expressing a positive willingness to pay: I want to preserve the seamount biodiversity for future generations. (Ison et al. 2021)	Time spent by a tourist in tree planting activities in or nearby the protected area because of their value of future uses of future generations.			Reason for expressing a positive willingness to pay: I want to preserve the seamount biodiversity for future generations. (Ison et al. 2021)
Learning	Learning value of the protected area for providing opportunities to learn about the environment expressed by a tourist measured as willingness to pay.		Learning value of the protected area for providing opportunities to learn about the environment expressed by a tourist measured as willingness to pay.	Mapping of education and learning: I can learn about the environment through scientific observation or experimentation. (Bogdan et al. 2019)<br><br>Mapping of inspiration value: It represents an inspiration source for		Mapping of education and learning: I can learn about the environment through scientific observation or experimentation. (Bogdan et al. 2019)<br><br>Mapping of inspiration value: It represents an inspiration source for	Mapping of education and learning: I can learn about the environment through scientific observation or experimentation. (Bogdan et al. 2019)<br><br>Mapping of inspiration value: It represents an inspiration source for

Valuation framework / Value type	TEV		TEEB		IPBES		
	Use	Non-use	Economic	Socio-cultural	Intrinsic	Relational	Instrumental
				folklore, artistic manifestations or national symbols. (Bogdan et al. 2019)		folklore, artistic manifestations or national symbols. (Bogdan et al. 2019)	folklore, artistic manifestations or national symbols. (Bogdan et al. 2019)
Social	The value of opportunities of visiting the protected area with friends and family expressed by a tourist as willingness to pay.	The value of opportunities for future generations to visit the protected area with friends and family expressed by a tourist as willingness to pay.	The value of opportunities of visiting the protected area with friends and family expressed by a tourist's willingness to pay.	Expression of the social value: *Protected area are important because they provide opportunities for social activities.* based on Likert scale. (Muñoz et al. 2019)	The value of biodiversity expressed by a tourist in terms of its right to exist.	Expression of the social value: *Protected area are important because they provide opportunities for social activities.* based on Likert scale. (Muñoz et al. 2019)	Expression of the social value: *Protected area are important because they provide opportunities for social activities* based on Likert scale. (Muñoz et al. 2019)
Tourism	Travel cost model. (Pongkijvorasin and Chotiyaputta 2013)		Travel cost model. (Pongkijvorasin and Chotiyaputta 2013)	Ecotourism value in terms of attraction through biodiversity and wildlife watching indicated on a Likert scale (Chakrabarty et al. 2019)		Preference for a protected area expressed by a tourist because touristic activities such as wildlife watching nurture their relationship with nature.	Travel cost model. (Pongkijvorasin and Chotiyaputta 2013)

Valuation framework / Value type	TEV		TEEB		IPBES		
	Use	Non-use	Economic	Socio-cultural	Intrinsic	Relational	Instrumental
Spiritual	Spiritual value of the protected area for providing sacred, religious, or spiritual activities expressed by a tourist measured as willingness to pay to participate in them.		Spiritual value of the protected area for providing sacred, religious, or spiritual activities expressed by a tourist measured as willingness to pay to use these places as tourist attractions.	Mapping of spiritual and religious values: It offers a special spiritual experience or it is the place where I feel a strong connection with nature. (Bogdan et al. 2019)<br><br>I value it because it is a specially spiritual place to me, or I feel the respect and reverence for nature there. (Chen et al. 2019)		Mapping of spiritual and religious values:<br>It offers a special spiritual experience or it is the place where I feel a strong connection with nature. (Bogdan et al. 2019)<br><br>I value it because it is a specially spiritual place to me, or I feel the respect and reverence for nature there. (Chen et al. 2019)	Spiritual value of the protected area for providing sacred, religious, or spiritual activities expressed by a tourist measured as willingness to pay to use these places as tourist attractions.
Indirect	Willingness to pay expressed by a tourist to preserve the Regulating Services / regulating Nature's Contributions to People in the protected area.		Willingness to pay expressed by a tourist to preserve the Regulating Services / regulating Nature's Contributions to People in the protected area.	Individual preferences to preserve Regulating Services / regulating nature's contributions to people, e.g., climate regulation or air purification.			Willingness to pay expressed by a tourist to preserve the Regulating Services / regulating Nature's Contributions to People in the protected area.
Conservation	Willingness to pay to conserve a particular species / habitat in the protected area for recreational use.	Willingness to pay to conserve a particular species / habitat for their right to exist.	Willingness to pay to conserve a particular species / habitat.	Use of recreational value based on social media content analysis to estimate conservation value. (Chakrabarty et al. 2019)	Conservation values as a measure of nature's value as an end in itself.	Conservation value of a protected area because it fosters human-nature connectedness and provides the space for social relationships among people.	Willingness to pay to conserve a particular species / habitat in the protected area for recreational use.

Valuation framework / Value type	TEV		TEEB		IPBES		
	Use	Non-use	Economic	Socio-cultural	Intrinsic	Relational	Instrumental
Historical			Historical value of places in the protected area expressed by a tourist measured as willingness to pay to use these places as tourist attractions.	I value it because it has places and things of natural and human history that matter to others, the nation, or me. (Chen et al. 2019)		I value it because it has places and things of natural and human history that matter to others, the nation, or me. (Chen et al. 2019)	Historical value of places in the protected area expressed by a tourist measured as willingness to pay to use these places as tourist attractions.
Relational				Expression of relational values on, e.g., observing nature; recreation and picnicking; historical and cultural values; education and science; local identity; spiritual values, based on Likert scale. (Mrotek et al. 2019)		Expression of relational values on, e.g., observing nature; recreation and picnicking; historical and cultural values; education and science; local identity; spiritual values, based on Likert scale. (Mrotek et al. 2019)	
Iconic		Willingness to pay for the conservation of the authenticity of the protected area. (Dagiliūtė et al. 2017)	Willingness to pay for the conservation of the authenticity of the protected area. (Dagiliūtė et al. 2017)	Preference assessment: This park represents a unique landscape found nowhere else on earth. (Baral et al. 2017)	The iconic value of a protected area expressed by tourists because the protected area has the right to be preserved because of its uniqueness.		
Thera-peutic	Therapeutic value of places in the protected area expressed by a tourist measured as willingness to pay for mental and physical		Therapeutic value of places in the protected area expressed by a tourist measured as willingness to pay for mental and physical health benefits gained through the visit.	I value it because it makes me feel better, physically and/or mentally. (Chen et al. 2019)		The therapeutic value of a protected area expressed by a tourist because the experience in the protected area nurtures them physically and/or mentally.	Therapeutic value of places in the protected area expressed by a tourist measured as willingness to pay for mental and physical health benefits gained through the visit.

Valuation framework / Value type	TEV		TEEB		IPBES		
	Use	Non-use	Economic	Socio-cultural	Intrinsic	Relational	Instrumental
	health benefits gained through the visit.						
Option (Use)	Willingness to pay for the maintenance of a protected area to travel to it in the future.		Willingness to pay for the maintenance of a protected area to travel to it in the future.	Time spent by a tourist in tree planting activities in or nearby the protected area because of their value of potential future uses.			Willingness to pay for the maintenance of a protected area to travel to it in the future.
Instrumental	Instrumental value of the protected area expressed by a tourist measured as willingness to pay for necessary food and supplies to sustain their life.	Donation to rainforest protection to contribute to the existence of wildlife for future generations to enjoy them.	Instrumental value of the protected area expressed by a tourist measured as willingness to pay for necessary food and supplies to sustain their life.	Expression of instrumental values on, e.g., providing potable water; food, firewood, or other economic resources, based on Likert scale. (Mrotek et al. 2019)			Expression of instrumental values on, e.g., providing potable water; food, firewood, or other economic resources, based on Likert scale. (Mrotek et al. 2019)
Local identity	Travel costs expressed by tourists to visit a protected area where ancestors lived.		Travel costs expressed by tourists to visit a protected area where ancestors lived.	Number of geo-tagged photographs regarding local identity, which included heritage, folklore, traditions, art and local workers (ranching, forestry, artisanal fishing, mining, and oil extraction). (Martínez Pastur et al. 2016)		The preference for a protected area expressed by tourist because it connects them with their ancestors.	Travel costs expressed by tourists to visit a protected area where ancestors lived.

References

Anna, Z., and D.S. Saputra. 2017. Economic valuation of whale shark tourism in Cenderawasih Bay National Park, Papua, Indonesia. *Biodiversitas Journal of Biological Diversity* 18: 1026–1034. doi:10.13057/biodiv/d180321.

Baral, N., H. Hazen, and B. Thapa. 2017. Visitor perceptions of World Heritage value at Sagarmatha (Mt. Everest) National Park, Nepal. *Journal of Sustainable Tourism* 25: 1494–1512. doi:10.1080/09669582.2017.1291647.

Bogdan, S.M., I. Stupariu, A. Andra-Topârceanu, and I.I. Năstase. 2019. Mapping social values for cultural ecosystem services in a mountain landscape in the Romanian Carpathians. *Carpathian Journal of Earth and Environmental Sciences* 14: 199–208. doi:10.26471/cjees/2019/014/072.

Castaño-Isaza, J., R. Newball, B. Roach, and W.W.Y. Lau. 2014. Valuing beaches to develop payment for ecosystem services schemes in Colombia's Seaflower marine protected area. *Ecosystem Services* 11: 22–31. doi:10.1016/j.ecoser.2014.10.003.

Chakrabarty, P., S. Pan, and R. Mandal. 2019. Promoting wildlife tourism on geotourism landscape: a study in Manas and Kaziranga National Parks of Assam, India. *GeoJournal of Tourism and Geosites* 24: 189–200. doi:10.30892/gtg.24115-352.

Chen, F., J. Wu, J. Liu, Y. Hu, X. Chen, P.-E. Lim, W.M. Aznan Abdullah, N.D. Mirah Sjafrie, et al. 2019. Comparison of social-value cognition based on different groups: The case of Pulau Payar in Malaysia and Gili Matra in Indonesia. *Ocean & Coastal Management* 173: 1–9. doi:10.1016/j.ocecoaman.2019.02.010.

Dagiliūtė, R., M. Žalandauskas, G. Sujetovienė, and J. Žaltauskaitė. 2017. Willingness to Pay for the Authenticity of the Curonian Spit. *Environmental Processes* 4: 251–262. doi:10.1007/s40710-017-0238-7.

Dehghani, M., P. Farshchi, A. Danekar, M. Karami, and A.A. Aleshikh. 2010. Recreation Value of Hara Biosphere Reserve using Willingness-to-pay method. *International Journal of Environmental Research* 4. doi:10.22059/ijer.2010.19.

Do, Y., and J.Y. Kim. 2020. An assessment of the aesthetic value of protected wetlands based on a photo content and its metadata. *Ecological Engineering* 150: 105816. doi:10.1016/j.ecoleng.2020.105816.

Heyes, C., and A. Heyes. 1999. Willingness to Pay Versus Willingness to Travel: Assessing the Recreational Benefits from Dartmoor National Park. *Journal of Agricultural Economics* 50: 124–139. doi:10.1111/j.1477-9552.1999.tb00799.x.

Ison, S., T. Ison, P. Marti-Puig, K. Needham, M.K. Tanner, and J.M. Roberts. 2021. Tourist Preferences for Seamount Conservation in the Galapagos Marine Reserve. *Frontiers in Marine Science* 7: 602767. doi:10.3389/fmars.2020.602767.

Martínez Pastur, G., P. L. Peri, M.V. Lencinas, M. García-Llorente, and B. Martín-López. 2016. Spatial patterns of cultural ecosystem services provision in Southern Patagonia. *Landscape Ecology* 31: 383–399. doi:10.1007/s10980-015-0254-9.

Mrotek, A., C.B. Anderson, A. E. Valenzuela, L. Manak, A. Weber, P. Van Aert, M. Malizia, and E.A. Nielsen. 2019. An evaluation of local, national and international perceptions of benefits and threats to nature in Tierra del Fuego National Park (Patagonia, Argentina). *Environmental Conservation* 46: 326–333. doi:10.1017/S0376892919000250.

Muñoz, L., V. Hausner, G. Brown, C. Runge, and P. Fauchald. 2019. Identifying spatial overlap in the values of locals, domestic- and international tourists to protected areas. *Tourism Management* 71: 259–271. doi:10.1016/j.tourman.2018.07.015.

Pongkijvorasin, S., and V. Chotiyaputta. 2013. Climate change and tourism: Impacts and responses. A case study of Khaoyai National Park. *Tourism Management Perspectives* 5: 10–17. doi:10.1016/j.tmp.2012.10.002.

Sinclair, M., M. Mayer, M. Woltering, and A. Ghermandi. 2020. Valuing nature-based recreation using a crowdsourced travel cost method: A comparison to onsite survey data and value transfer. *Ecosystem Services* 45: 101165. doi:10.1016/j.ecoser.2020.101165.

IUCN and UNEP-WCMC. 2021. The World Database on Protected Areas (WDPA) [On-line], [December 2021]. Cambridge, UK: UNEP-WCMC. Available at: www.protectedplanet.net.

Viirret, E., K. Raatikainen, N. Fagerholm, N. Käyhkö, and P. Vihervaara. 2019. Ecosystem Services at the Archipelago Sea Biosphere Reserve in Finland: A Visitor Perspective. *Sustainability* 11: 421. doi:10.3390/su11020421.
